# Lamella-nanostructured eutectic zinc–aluminum alloys as reversible and dendrite-free anodes for aqueous rechargeable batteries

**DOI:** 10.1038/s41467-020-15478-4

**Published:** 2020-04-02

**Authors:** Sheng-Bo Wang, Qing Ran, Rui-Qi Yao, Hang Shi, Zi Wen, Ming Zhao, Xing-You Lang, Qing Jiang

**Affiliations:** Key Laboratory of Automobile Materials (Jilin University), Ministry of Education, and School of Materials Science and Engineering, Jilin University, Changchun, 130022 China

**Keywords:** Batteries, Materials for energy and catalysis

## Abstract

Metallic zinc is an attractive anode material for aqueous rechargeable batteries because of its high theoretical capacity and low cost. However, state-of-the-art zinc anodes suffer from low coulombic efficiency and severe dendrite growth during stripping/plating processes, hampering their practical applications. Here we show that eutectic-composition alloying of zinc and aluminum as an effective strategy substantially tackles these irreversibility issues by making use of their lamellar structure, composed of alternating zinc and aluminum nanolamellas. The lamellar nanostructure not only promotes zinc stripping from precursor eutectic Zn_88_Al_12_ (at%) alloys, but produces core/shell aluminum/aluminum sesquioxide interlamellar nanopatterns in situ to in turn guide subsequent growth of zinc, enabling dendrite-free zinc stripping/plating for more than 2000 h in oxygen-absent aqueous electrolyte. These outstanding electrochemical properties enlist zinc-ion batteries constructed with Zn_88_Al_12_ alloy anode and K_*x*_MnO_2_ cathode to deliver high-density energy at high levels of electrical power and retain 100% capacity after 200 hours.

## Abstract (web summary)

Aqueous rechargeable Zn-ion batteries are attractive energy storage devices, but their wide adoption is impeded by the irreversible metallic Zn anode. Here the authors report lamellar-nanostructured eutectic Zn/Al alloys as reversible and dendrite-free anodes for improved battery performance.

## Introduction

Widespread utilization of plentiful but only intermittently available solar and wind power has raised urgent demand for the development of safe, cost-effective, and reliable grid-scale energy storage technologies for efficient integration of renewable energy sources^1,2^. Among many electrochemical energy storage technologies, rechargeable battery based on Zn metal chemistry in neutral aqueous electrolyte is one of the most attractive devices by virtue of metallic Zn having high volumetric and gravimetric capacity (5854 mAh cm^−3^ and 820 mAh  g^−1^), low Zn/Zn^2+^ redox potential (−0.76 V versus standard hydrogen electrode), high abundance and low cost^3,4^. Along with high ionic conductivities (up to 1 S cm^−1^) of aqueous electrolytes and two-electron redox reaction of Zn/Zn^2+^ that favor high rate capability and high energy density, respectively, aqueous rechargeable Zn-ion batteries (AR-ZIBs) promise safe and low-cost high-density energy storage/delivery at fast charge/discharge rates for stationary grid storage applications^5,6^. This has prompted the recent renaissance of AR-ZIBs^4,7,8^, with the development of various cathode materials including polymorphous manganese dioxides^9–13^, vanadium oxides^14–19^, Prussian blue analogues (PBAs)^20,21^ and quinone analogs^22^ for hosting/delivering Zn^2+^ and/or H^+^ via insertion/extraction or chemical conversion reactions^23–25^. However, no matter which advanced material is employed as the cathode, state-of-the-art AR-ZIBs are persistently plagued by the irreversibility issues of traditional metallic Zn anode^5,6,8,26^, such as dendrite formation and growth^5,6,8,27,28^ and low coulombic efficiency (CE) associated with side reactions (e.g., hydrogen evolution, corrosion, and by-product formation) during the stripping/plating processes^29–31^. Although the Zn dendrite formation could be effectively alleviated in neutral electrolytes compared with in alkaline solutions^7–9^, it is inherently unavoidable because of the unique metallurgic characteristics of monometallic Zn^27,31^. Furthermore, there always take place uncontrollable shape changes to produce abundant cracks or defects in the repeated processes of Zn stripping/plating^32,33^. The structural irreversibility triggers further Zn dendrite growth due to uneven distribution and slow diffusion of Zn^2+^ ions at the Zn metal/electrolyte interface^33^ and continuously depletes Zn and electrolyte via supplementary side reactions^30,31^, leading to rapid and remarkable capacity fading and short lifespan of AR-ZIBs. Therefore, it is highly desirable to explore novel Zn-based anode materials that can circumvent these irreversibility issues for constructing high-performance AR-ZIBs.

Here we report that a class of eutectic Zn/Al alloys with an alternating Zn and Al lamellar nanostructure as reversible and dendrite-free anode materials significantly improve electrochemical performance of aqueous rechargeable zinc-manganese oxide batteries (Zn-Mn AR-ZIBs). The unique lamellar structure promotes the reversibility of stripping/plating of Zn by making use of symbiotic less-noble Al lamellas, which in-situ form interlamellar nanopatterns with an Al/Al_2_O_3_ core/shell structure. Therein, the Al protects against irreversible by-product of ZnO or Zn(OH)_2_ while the insulating Al_2_O_3_ shell prevents the electro-reduction of Zn^2+^ ions on the Al/Al_2_O_3_ patterns and thus guides their electrodeposition on the precursor Zn sites, substantially eliminating the formation and growth of Zn dendrites. As a result, the eutectic Zn_88_Al_12_ (at%) alloys exhibit superior dendrite-free Zn stripping/plating behaviors, with remarkably low and stable overpotential, for more than 2000 h in O_2_-absent aqueous ZnSO_4_ electrolyte. The outstanding electrochemical properties enable the Zn-Mn AR-ZIBs constructed with eutectic Zn_88_Al_12_ alloy anode and K_*x*_MnO_2_ cathode to deliver energy density of ∼230 Wh kg^−1^ (based on the mass of K_*x*_MnO_2_ cathode) at high levels of electrical power while retaining ∼100% capacity after more than 200 hours. By adjusting the anode-to-cathode mass ratio to 3:1, the overall energy density of Zn-Mn AR-ZIB can reach ∼142 Wh kg^−1^ based on total mass of anode and cathode. The strategy of eutectic-composition alloying could open an avenue to the development of high-performance metallic anodes for next-generation secondary batteries.

## Results

### Eutectic alloying strategy for Zn dendrite suppression

**Fig. 1:**
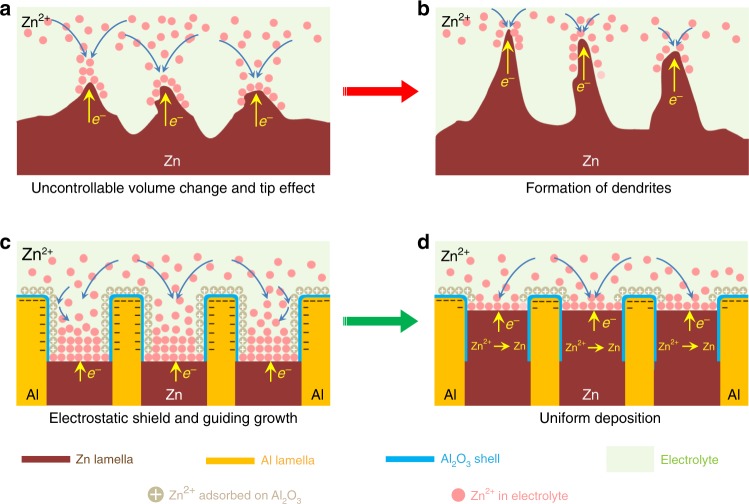
Schematic illustration of eutectic strategy for dendrite and crack suppression. **a** Monometallic Zn electrodes with abundant cracks or defects that are produced by uncontrollable volume change in the Zn stripping/plating processes. **b** Growth of Zn dendrites triggered by uncontrollable volume change and tip effect. **c** Eutectic Zn/Al alloys with a lamellar structure composed of alternative Zn and Al nanolamellas in-situ produce core/shell interlayer patterns during the Zn stripping to guide the subsequent Zn plating. **d** The Al/Al_2_O_3_ interlayer patterns associated with insulative Al_2_O_3_ shield facilitate the uniform deposition of Zn.

Zn metal is a classic anode material but works as a hostless electrode to store/deliver energy via the electrochemical plating/stripping of Zn, during which the Zn^2+^ cations thermodynamically prefer to form nuclei at the dislocated sites and grow into initial protuberances on the surface of Zn substrate with uncontrollable Zn redistribution (Fig. 1a)^27–29,31,33^. In particular, the tips of protuberances not only have higher potentials^34^ but consist of high-density low-coordination steps and kinks with lower activation energy, both of which facilitate further growth of dendrites (Fig. 1b)^29^. To circumvent these irreversibility problems, here we propose an eutectic-composition alloying strategy based on Zn/Al alloy system, wherein the eutectic structure is composed of alternating Zn and Al lamellas. Although the standard equilibrium potential of Al^3+^/Al (−1.66 V versus SHE) is much lower than that of Zn^2+^/Zn^35^, the formation of Al_2_O_3_ shell on the Al lamellas protects against the dissolution of Al and thus allows the selectively electrochemical stripping/plating of Zn in aqueous electrolyte^35,36^. Their distinct electrochemical behaviors enable the different roles of Zn and Al lamellas in the charge/discharge processes: the former supplying Zn^2+^ charge carriers and the latter serving as 2D hosting skeleton to accommodate the Zn plating (Fig. 1c). Owing to the insulating Al_2_O_3_ shell that substantially blocks the electron transfer from Al to the Zn^2+^ cations^35^, there forms a positive electrostatic shield around the Al/Al_2_O_3_ lamellas without the reduction of Zn^2+^ ^37^, enlisting the Al/Al_2_O_3_ nanopatterns to guide the uniform Zn deposition at their interlayer spacing along the Zn precursor sites (Fig. 1d).

### Preparation and characterization of eutectic Zn-Al alloys

**Fig. 2:**
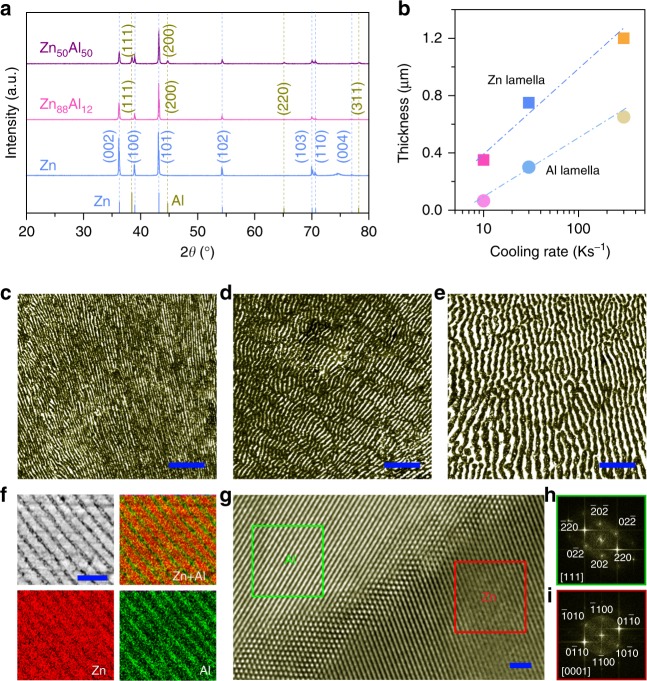
Microstructure characterization of eutectic Zn/Al alloys. **a** XRD patterns of monometallic Zn, hypoeutectic Zn_50_Al_50_ and eutectic Zn_88_Al_12_ alloys. The line patterns show reference cards 04-0831 for hcp Zn (blue) and 04-0787 for fcc Al (dark yellow) according to JCPDS. **b** Thickness of Zn and Al layers in lamella-nanostructured eutectic Zn_88_Al_12_ alloys that are produced at various cooling rates. **c**–**e** Optical micrographs of lamella-nanostructured eutectic Zn_88_Al_12_ alloys with lamella spacing of ∼450 nm (**c**), ∼1050 nm (**d**) and ∼1850 nm (**e**). Scale bar, 10 μm (**c**–**e**), Typical SEM image lamella-nanostructured eutectic Zn_88_Al_12_ alloys with lamella spacing of ∼450 nm and the corresponding EDS element mapping of Zn and Al. Scale bar, 2 μm. **g**, HRTEM image of Zn/Al interface of eutectic Zn_88_Al_12_ alloys (*λ* = ∼450 nm). Scale bar, 1 nm. **h**, **i**, FFT patterns of selected areas of HRTEM image (**g**) that correspond to fcc Al (**h**) and hcp Zn (**i**), respectively.

Eutectic Zn_88_Al_12_ (at%) alloys are produced by a facile and scalable metallurgic procedure, viz. alloying pure Zn and Al metals and pouring casting at various cooling rates from ∼10 to ∼300 K s^−1^. Supplementary Fig. 1 shows typical X-ray diffraction (XRD) patterns of eutectic Zn_88_Al_12_ alloys, with the major peaks corresponding to the primary hexagonal closest packed (hcp) Zn phase (JCPDS 04-0831), apart from the weak ones attributed to the face-centered cubic (fcc) α-Al phase (JCPDS 04-0787) (Fig. 2a). Distinguished from hypoeutectic Zn_50_Al_50_ alloy that is composed of random eutectic mixtures of Zn and Al (Supplementary Figs. 2 and 3)^38^, the eutectic Zn_88_Al_12_ alloys exhibit an ordered lamellar structure of alternating Zn and Al lamellas. As a result of the rapid solidification triggered Al phase precipitation as well as the balance between the lateral diffusion of excess Zn and Al in the liquid just ahead of the solid/liquid interface and the creation of Zn/Al interfacial area during the solidification process^39,40^, the thickness of Zn or Al lamellas, or the interlamellar spacing (λ), decreases with the cooling rates (Fig. 2b). Figure 2c–e show representative optical micrographs of the lamella-structured eutectic Zn_88_Al_12_ alloys, which are prepared at the cooling rates of ∼10, ∼30 and ∼300 K s^−1^, respectively. At the slow cooling rate of ∼10 K s^−1^, the λ of the eutectic Zn_88_Al_12_ alloy is ∼450 nm (Fig. 2c and Supplementary Fig. 4a), i.e., ∼350 nm thick Zn lamellas (sagging stripes) alternatingly sandwiched by the Al ones (protruding stripes) with thickness of ∼100 nm (Supplementary Fig. 5). The unique lamellar structure is further illustrated by scanning electron microscope (SEM) backscattered electron image and the corresponding energy-dispersive X-ray spectroscopy (EDS) elemental mappings, with the uniform distribution of alternating Zn and Al lamellas (Fig. 2f). While increasing the cooling rate to ∼300 K s^−1^, the λ reaches ∼1850 nm, with ∼1200-nm-thick Zn lamellas and ∼650-nm-thick Al lamellas (Fig. 2e and Supplementary Fig. 4c). Figure 2g shows a typical high-resolution transmission electron microscope (HRTEM) image of Zn/Al interfacial region, demonstrating the symbiotic Zn and Al lamellas viewed along their 〈0001〉 and 〈111〉 zone axis. The fast Fourier transform (FFT) patterns of the selected areas in Fig. 2g confirm the fcc Al phase (Fig. 2h) and the hcp Zn phase (Fig. 2i) separated from each other during the solidification process^39,40^.

**Fig. 3:**
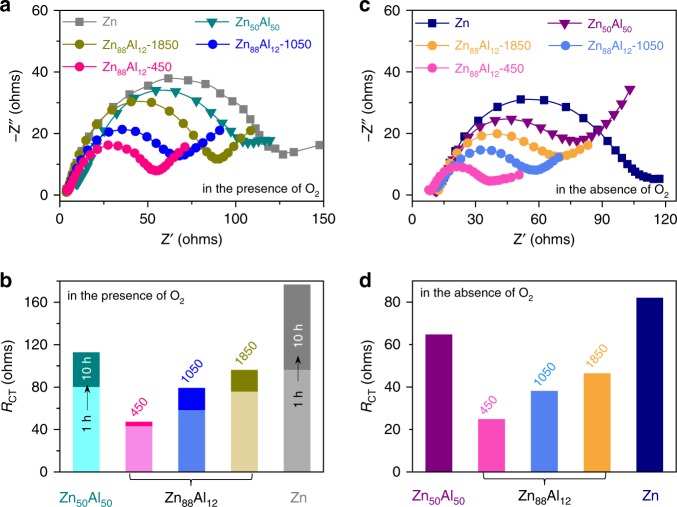
Oxidation-resistance capability of Zn metal and eutectic Zn/Al alloys. **a** Electrochemical impedance spectra (EIS) of eutectic Zn_88_Al_12_ alloys with various lamellar spacings (*λ* = ∼450, ∼1050 and ∼1850 nm), hypoeutectic Zn_50_Al_50_ alloy and monometallic Zn after immersed in the O_2_-present ZnSO_4_ aqueous electrolytes for 1 h. **b** Evolutions of the charge transfer resistances (*R*_CT_) of eutectic Zn_88_Al_12_ alloys with various lamellar spacings (*λ* = ∼450, ∼1050 and ∼1850 nm), hypoeutectic Zn_50_Al_50_ alloy and monometallic Zn when extending the immersing time from 1 to 10 h in the O_2_-present ZnSO_4_ aqueous electrolytes. **c**, **d** EIS spectra of eutectic Zn_88_Al_12_ alloys with various lamellar spacings (*λ* = ∼450, ∼1050 and ∼1850 nm), hypoeutectic Zn_50_Al_50_ alloy and monometallic Zn (**c**) and their corresponding *R*_CT_ values (**d**) in the O_2_-absent ZnSO_4_ aqueous electrolyte for 1 h.

Despite the immiscibility of Zn and Al metals, the lamella-structured eutectic Zn_88_Al_12_ exhibits remarkable alloy nature, with a superior oxidation-resistance capability in air and aqueous electrolytes compared with monometallic Zn, because of the formation of stable and passive Al_2_O_3_ surface layer, which protects against the further oxidation^39,40^. As shown in optical photographs (Supplementary Fig. 6a), the eutectic Zn_88_Al_12_ alloy still displays a metallic lustre after exposed to air for five days, in sharp contrast with monometallic Zn that undergoes severe oxidation. Furthermore, the thinner the interlamellar spacing, the higher the oxidation-resistance capability. Even when immersing in the O_2_-present ZnSO_4_ aqueous electrolyte for 72 h, the eutectic Zn_88_Al_12_ alloy with *λ* = ∼450 nm does not display evident change (Supplementary Fig. 6b). The superior oxidation-resistance behavior of eutectic Zn_88_Al_12_ alloys is further demonstrated by their EIS measurements, which are performed on the basis of a classic three-electrode configuration with Pt foil as the counter electrode and an Ag/AgCl electrode as the reference electrode, in the O_2_-present ZnSO_4_ electrolyte (Fig. 3a and Supplementary Fig. 7b). In the Nyquist plot, the EIS spectra of eutectic Zn_88_Al_12_ alloys, hypoeutectic Zn_50_Al_50_ alloy and monometallic Zn display characteristic semicircles with distinct diameters in the high- and middle-frequency range. At high frequencies, the intercept at the real part represents the intrinsic resistance of both electrolyte and electrode (*R*_I_); in the middle-frequency range, the diameter of semicircle corresponds to the charge transfer resistance (*R*_CT_) and the double-layer capacitance (*C*_F_); and the slope of the inclined line at flow frequencies is the Warburg resistance (*Z*w). Based on the equivalent circuit with these general descriptors (Supplementary Fig. 7a), the EIS spectra are analyzed using the complex nonlinear least-squares fitting method. Supplementary Fig. 7c compares the *R*_I_ values of all Zn-based electrodes immersed in the O_2_-present electrolyte for 1 h, wherein the Zn_88_Al_12_ with λ = ∼450 nm has the lowest *R*_I_ value (~11 Ω) because of the outstanding oxidation-resistance property. Even extending the immersion time to 10 h, the Zn_88_Al_12_ still maintains ∼11 Ω whereas the Zn electrode has the *R*_I_ value to increase to ∼22 Ω from ∼18 Ω. The large change of *R*_I_ value indicates the inferior oxidation-resistance capability of the monometallic Zn. Owing to their different oxidation-resistance capabilities, there form distinct oxide layers to depress the Zn stripping/plating kinetics, indicated by the increase of *R*_CT_ value. When immersed in the O_2_-present electrolyte for 1 and 10 h, the Zn_88_Al_12_ with *λ* = ∼450 nm exhibits exceptional stability with the *R*_CT_ value changing from ∼32 Ω to ∼36 Ω, in sharp contrast with the monometallic Zn electrode with a remarkable change of *R*_CT_ from ∼96 Ω to ∼177 Ω (Fig. 3b). This is probably because there lacks a passivation film on the Zn lamella surface in virtue of the protection of neighboring Al lamellas^5,26^. More impressively, the superior oxidation-resistance capability enlists the eutectic Zn_88_Al_12_ alloys to be more conducive to electron transfer during the electrochemical Zn stripping/plating processes in the O_2_-absent ZnSO_4_ aqueous electrolyte. As demonstrated by EIS spectra in Fig. 3c, the eutectic Zn_88_Al_12_ with *λ* = ∼450 nm has the *R*_I_ and *R*_CT_ values of as low as ∼9 Ω and ∼24  Ω, respectively (Fig. 3d and Supplementary Fig. 7e). Although the increase of *λ* may weaken the protecting effect of Al on the eutectic Zn_88_Al_12_, the value of *R*_CT_ is only about half of that of the monometallic Zn (~82 Ω) (Fig. 3d and Supplementary Fig. 8).

### Electrochemical properties of eutectic Zn_88_Al_12_ alloys

**Fig. 4:**
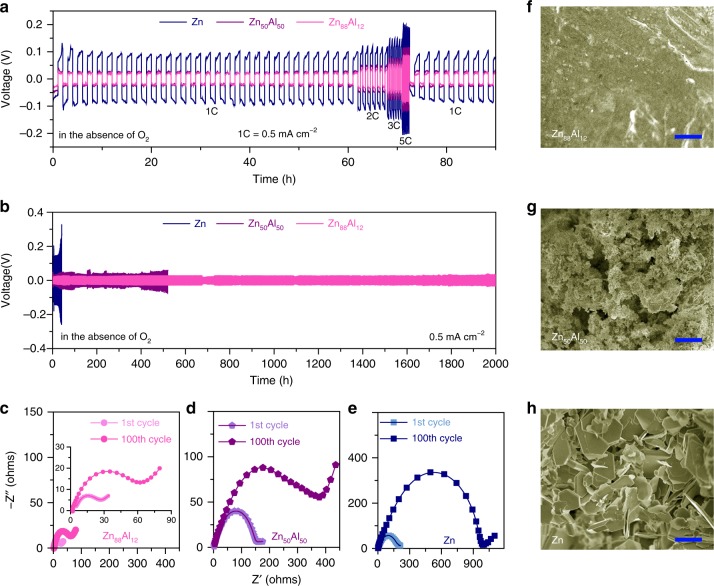
Electrochemical performance of symmetric batteries of Zn or Zn-Al alloy electrodes. **a** Comparison of voltage profiles for monometallic Zn, hypoeutectic Zn_50_Al_50_ and eutectic Zn_88_Al_12_ (*λ* = ∼450 nm) symmetric batteries during Zn stripping/plating at various current densities from 1 to 5 C in aqueous ZnSO_4_ electrolyte with the absence of O_2_, where 1 C = 0.5 mA cm^−2^. **b** Long-term Zn stripping/plating cycling of symmetric batteries of monometallic Zn, hypoeutectic Zn_50_Al_50_ or eutectic Zn_88_Al_12_ alloys (*λ* = ∼450 nm) at the current density of 0.5 mA cm^−2^ in aqueous ZnSO_4_ electrolyte with the absence of O_2_. **c**–**e** Comparisons of EIS spectra for eutectic Zn_88_Al_12_ (*λ* = ∼450 nm) (**c**), hypoeutectic Zn_50_Al_50_ (**d**) monometallic Zn (**e**) symmetric batteries after the 1^st^ and 100^th^ cycles in aqueous ZnSO_4_ electrolyte in the absence of O_2_. Inset: Expanded view for EIS of Zn_88_Al_12_. **f**–**h** SEM images of eutectic Zn_88_Al_12_ (*λ* = ∼450 nm) (**f**), hypoeutectic Zn_50_Al_50_ (**g**) monometallic Zn (**h**), electrodes after long-term Zn stripping/plating cycling measurements for 2000, 520, and 42 h in aqueous ZnSO_4_ electrolyte with the absence of O_2_, respectively. Scale bare, 5 μm (**f**–**h**).

To investigate the Zn stripping/plating behaviors of the Zn-based electrodes, electrochemical measurements are performed on symmetric batteries that are constructed with two identical electrodes. Figure 4a shows the voltage profiles of the eutectic Zn_88_Al_12_ symmetric battery during the Zn plating/stripping processes at various current densities in the O_2_-absent ZnSO_4_ electrolyte, comparing with those of the hypoeutectic Zn_50_Al_50_ and monometallic Zn ones. The battery based on the eutectic Zn_88_Al_12_ alloy with *λ* = ∼450 nm exhibits a relatively flat and stable voltage plateau with the absolute overpotential of ∼20 mV at the rate of 1C (where 1C represents a one-hour complete charge or discharge at the current density of 0.5 mA cm^−2^), much lower than the value of symmetric Zn battery (~101 mV). The less polarization is probably due to the unique eutectic structure of alternating Zn and Al lamellas in the Zn_88_Al_12_ alloy. Therein, the constituent Al lamellas not only protect against the passivation of the electroactive Zn but reduce the local current density of Zn stripping/plating via the formation of core/shell Al/Al_2_O_3_ lamellar nanopatterns (Supplementary Fig. 9a)^41,42^, which guide the uniform Zn electrodeposition in the subsequent plating process (Supplementary Fig. 9b). During the Zn stripping/plating, the XRD and Raman spectroscopy characterizations evidence the absence of passivation film on the electroactive Zn lamellas of Zn_88_Al_12_ (Supplementary Fig. 10a, c), which usually forms on the monometallic Zn electrode. As shown in Supplementary Fig. 10b, d for the Zn electrode after cycling test, there appear neoformative diffraction peaks and characteristic Raman bands corresponding to Zn_4_SO_4_(OH)_6_·H_2_O in addition to ZnO^4,8,11,43^. These observations are in agreement with surface chemical states of Zn or/and Al, which are analyzed by X-ray photoelectron spectroscopy (XPS). After cycling test, the surface Zn of monometallic Zn electrode is completely oxidized because of the formation of Zn_4_SO_4_(OH)_6_·H_2_O and ZnO (Supplementary Fig. 11a), different from that of the pristine one with primary metallic Zn^0^ in addition to some Zn^2+^ due to the initial surface oxidation (Supplementary Fig. 11b). While for the Zn_88_Al_12_ electrode after cycling test, the Zn 2p and Al 2p XPS spectra reveal that the surface Zn maintains almost the same chemical states as that in the pristine one (Supplementary Fig. 11c, e), but the metallic Al mainly becomes Al^3+^ as a consequence of the formation of Al_2_O_3_ shell (Supplementary Fig. 11d, f). As the stripping/plating rate increases to 5C, the overpotential of the symmetric Zn_88_Al_12_ battery only increases to ∼82 mV, implying the excellent rate capability of eutectic Zn_88_Al_12_ alloy electrode. The high reversibility of Zn stripping/plating on the eutectic Zn_88_Al_12_ alloy electrode is further attested by chronocoulometry measurements based on a three-electrode cell, in which the Zn electrodes are employed as the reference and counter electrodes (inset of Supplementary Fig. 12). The Zn stripping/plating on the eutectic Zn_88_Al_12_ alloy is highly reversible, with the CE of ∼100 %, during the cycling test for more than 100 cycles (Supplementary Fig. 12).

During a long-term Zn stripping/plating cycling measurement, the voltage profile of Zn_88_Al_12_ battery does not display any evident voltage hysteresis or change even for more than 2000 h, in sharp contrast to those of the Zn_50_Al_50_ and Zn ones with much larger voltage hysteresis and fluctuation after 100 and 26 hours, respectively (Fig. 4b). Specifically, there takes place an abrupt voltage drop after a dramatic voltage increase in the Zn battery, which is caused by a short circuit of battery due to the formation of Zn dendrites. EIS spectra also justify the outstanding stability of the Zn_88_Al_12_ alloy electrode during the Zn stripping/plating processes because of the unique eutectic structure (Fig. 4c–e). Furthermore, the fact that inductively coupled plasma optical emission spectroscopy (ICP-OES) cannot detect Al^3+^ ions in the O_2_-absent aqueous electrolytes demonstrates the chemical stability of Al/Al_2_O_3_ interlamellar nanopatterns (Supplementary Table 1), which in turn guide the deposition of Zn after a long-term cycling test of the Zn_88_Al_12_. As shown in Fig. 4f, the eutectic Zn_88_Al_12_ alloy electrode still keeps a smooth surface after more than 1000 cycles of Zn stripping/plating. This is distinctly distinguished from the cycled hypoeutectic Zn_50_Al_50_ and monometallic Zn electrodes even in fewer cycles, wherein the former displays an uneven porous structure (Fig. 4g) and the latter undergoes severe growth of dendrites and cracks (Fig. 4h). The addition of Mn^2+^ ions in the aqueous ZnSO_4_ electrolyte does not remarkably influence the Zn stripping/plating behavior of Zn_88_Al_12_ alloy. As shown in Supplementary Fig. 13, the Zn_88_Al_12_ battery exhibits almost the same voltage-time profiles in the 2 M ZnSO_4_ electrolyte without/with 0.2 M MnSO_4_. While in the ZnSO_4_ electrolyte with the O_2_ concentration of 16.59 mg L^−1^, the eutectic Zn_88_Al_12_ battery exhibits a stable voltage profile for more than 400 hours (Supplementary Fig. 14a), followed by slightly increasing voltage hysteresis due to the morphology evolution probably triggered by the partial oxidation of Zn via the reactions (Supplementary Fig. 14b)^8,43^: Zn_88_Al_12_ + O_2_ + H_2_O → Al_2_O_3_ + Zn(OH)_2_ + Zn^2+^ + e^−^ and Zn(OH)_2_ + 2e^−^ → ZnO + H_2_O^11^. Nevertheless, the lamellar structure of alternating Zn and Al lamellas significantly alleviate structure changes, in comparison with the electrodes of hypoeutectic Zn_50_Al_50_ alloy and monometallic Zn (Supplementary Fig. 14c–e).

### Electrochemical performance of Zn-ion full batteries

In view of the outstanding electrochemical properties, the lamella-structured eutectic Zn_88_Al_12_ alloy with *λ* = ∼450 nm is used as the anode to couple with potassium manganese oxide (K_*x*_MnO_2_) cathode material for demonstrating its actual application in Zn-ion full batteries, with an aqueous electrolyte containing 2 M ZnSO_4_ and 0.2 M MnSO_4_. Therein, tetragonal α-K_*x*_MnO_2_ nanofibers are synthesized by a stirring hydrothermal approach (Supplementary Fig. 15)^44^. Supplementary Fig. 16a shows typical cyclic voltammetry (CV) curves of Zn_88_Al_12_/K_*x*_MnO_2_ full battery in the aqueous electrolytes, without and with the presence of O_2_, exhibiting a similar Zn storage/delivery behavior with well-defined redox peaks during the charge/discharge processes^4,7,8,10–12^. It implies that the electrolyte in the absence of O_2_ does not substantially change the Zn^2+^ (de-)intercalation mechanism within the K_*x*_MnO_2_, i.e., δZn^2+^ + 2δe^−^ + K_*x*_MnO_2_ ↔ δZnK_*x*_MnO_2_^4,7,8,10–12^, except for boosting the reaction kinetics of Zn stripping/plating due to the absence of passivation oxide (e.g., ZnO or Zn(OH)_2_) on the Zn lamella surface of the Zn_88_Al_12_ (Supplementary Fig. 16b).

**Fig. 5:**
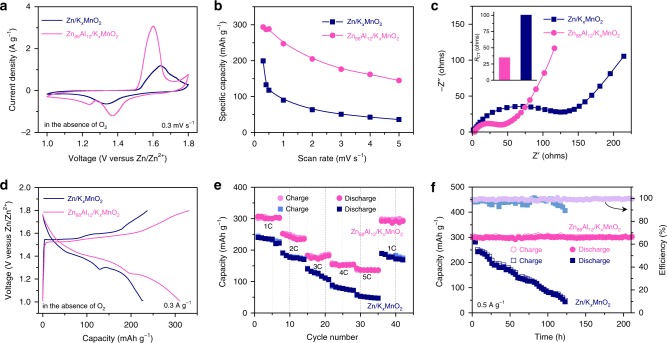
Electrochemical performance of zinc-ion full batteries. **a** Typical CV curves for Zn_88_Al_12_/K_*x*_MnO_2_ and Zn/K_*x*_MnO_2_ batteries, which are constructed with the K_*x*_MnO_2_ nanofibers as the cathode and the eutectic Zn_88_Al_12_ alloy (*λ* = ∼450 nm) or the monometallic Zn as the anode, in the O_2_-absent ZnSO_4_ aqueous electrolyte. Scan rate: 0.3 mV s^−1^. **b** Specific capacities for Zn_88_Al_12_/K_*x*_MnO_2_ and Zn/K_*x*_MnO_2_ batteries at various scan rates. **c** EIS spectra of Zn_88_Al_12_/K_*x*_MnO_2_ and Zn/K_x_MnO_2_ batteries and their corresponding *R*_CT_ values (inset) in the O_2_-absent ZnSO_4_ aqueous electrolyte. **d** Typical voltage profiles of Zn_88_Al_12_/K_*x*_MnO_2_ and Zn/K_*x*_MnO_2_ batteries at the charge/discharge current density of 0.3 A g^−1^. **e** Comparison for rate capabilities of Zn_88_Al_12_/K_*x*_MnO_2_ and Zn/K_*x*_MnO_2_ batteries at various rates from 1 to 5 C. **f** Capacity retention and coulombic efficiency of the Zn_88_Al_12_/K_*x*_MnO_2_ battery in a long-term cycling test at 0.5 A g^−1^, comparing with those of the Zn/K_*x*_MnO_2_ battery.

Figure 5a compares representative CV curve of Zn-ion batteries that are constructed with the K_*x*_MnO_2_ cathode and the Zn_88_Al_12_ or Zn anode, in the O_2_-absent aqueous electrolyte. The use of different anode materials, i.e., the lamella-structured eutectic Zn_88_Al_12_ alloy and the single-phase structured monometallic Zn, enlists them to exhibit distinct voltammetric behaviors. Relative to the Zn/K_*x*_MnO_2_ battery, the Zn_88_Al_12_/K_*x*_MnO_2_ has remarkably enhanced current density and shifts anodic/cathodic peaks to more negative/positive voltages, respectively, indicating that the Zn_88_Al_12_ is more conducive to the Zn storage/delivery than the Zn^4,7,8,18^. As a result, the Zn_88_Al_12_/K_*x*_MnO_2_ battery exhibits a superior rate capability in the scan rates from 0.3 to 5 mV s^−1^ (Supplementary Fig. 17a, b). As shown in Fig. 5b, the Zn_88_Al_12_/K_*x*_MnO_2_ achieves a specific capacity of as high as ∼294 mAh g^−1^ at 0.3 mV s^−1^. Even when the scan rate is increased to 5 mV s^−1^ (i.e., the discharge time of 160 s), it still retains the capacity of ∼145 mAh g^−1^, about four-fold higher than the value of the Zn/K_*x*_MnO_2_ battery (~36 mAh g^−1^). The expectation that the lamella-structured eutectic Zn_88_Al_12_ alloy ameliorates the kinetics of Zn strippling/plating is further verified by the EIS analysis (Fig. 5c), with the *R*_CT_ value of the Zn_88_Al_12_/K_*x*_MnO_2_ being ∼66 Ω lower than that of the Zn/K_*x*_MnO_2_ (inset of Fig. 5c). Figure 5d presents typical voltage profiles for the charge/discharge processes of Zn_88_Al_12_/K_*x*_MnO_2_ and Zn/K_*x*_MnO_2_ batteries at a current density of 0.3 A g^−1^, with the plateaus that are consistent with the redox peaks in the CV curves shown in Fig. 5a. Because of the improved Zn stripping/plating in the eutectic Zn_88_Al_12_ anode, the Zn_88_Al_12_/K_*x*_MnO_2_ evidently outperforms the Zn/K_*x*_MnO_2_ at various charge/discharge rates (Fig. 5e and Supplementary Fig. 18). As shown in the Ragone plot, the energy densities of Zn_88_Al_12_/K_*x*_MnO_2_ battery, based on the mass of K_*x*_MnO_2_ cathode, reaches ∼230 Wh kg^−1^, more than four-fold higher than the value of Zn/K_*x*_MnO_2_ at the electrical power of ∼550 kW kg^−1^. Based on the total mass of anode and cathode in the full Zn_88_Al_12_/K_*x*_MnO_2_ battery, the overall energy density can reach ∼142 Wh kg^−1^ by lowering the anode-to-cathode mass ratio to 3:1 (Supplementary Fig. 19). Supplementary Fig. 20 shows the self-discharge performance of the Zn_88_Al_12_/K_*x*_MnO_2_ battery. In the O_2_-absent electrolyte, the voltage of Zn_88_Al_12_/K_*x*_MnO_2_ battery drops to 1.481 V in ∼13 h, slower than the one with the O_2_-present electrolyte, of which the voltage decreases to 1.472 V in ∼6 h. The evident voltage drop is due to the pseudocapacitive discharge behavior, which is probably boosted by the presence of O_2_. While in the subsequent 600 h, the Zn_88_Al_12_/K_*x*_MnO_2_ batteries with the O_2_-present and O_2_-absent electrolytes exhibit a voltage plateau with very low self-discharge (~0.1 mV h^−1^) because of ultralow insertion kinetics of Zn^2+^ ^23–25,43^. The cycling life of Zn_88_Al_12_/K_*x*_MnO_2_ batteries is tested by galvanostatic charge/discharge at current densities of 0.5 and 5 A g^−1^, respectively (Fig. 5f and Supplementary Fig. 21). The significant capacitance retention, about 100% of the initial capacitance after more than 200 h or 5000 cycles, indicates its impressive long-term durability with nearly 100% efficiency in the voltage window between 1.0 and 1.8 V. In sharp contrast, the Zn/K_*x*_MnO_2_ battery undergoes fast capacity degradation (Fig. 5f). This probably results from the irreversibility issues of monometallic Zn, i.e., the dendrite formation and growth associated with side reactions, in view that the K_*x*_MnO_2_ cathode still maintains the initial morphology and crystallographic structure after the cycling measurement (Supplementary Fig. 22).

## Discussion

In summary, we have proposed eutectic-composition alloying, based on the Zn_88_Al_12_ alloy with a lamellar structure composed of alternating Zn and Al nanolamellas, as an effective strategy to tackle irreversibility issues of Zn metal anode caused by the growth of dendrites and cracks during the stripping/plating processes. By virtue of symbiotic less-noble Al lamellas, which not only protects the constituent Zn lamellas from the formation of irreversible ZnO or Zn(OH)_2_ by-product but also in-situ form stable Al/Al_2_O_3_ interlamellar patterns during the Zn stripping and in turn guide subsequent growth of Zn, the eutectic Zn_88_Al_12_ (at%) alloys exhibit superior dendrite-free Zn stripping/plating behaviors, with low overpotential and high coulombic efficiency, for more than 2000 h in O_2_-absent aqueous ZnSO_4_ electrolyte. The use of the eutectic Zn_88_Al_12_ alloy as the anode enlists the Zn-ion full batteries with the K_*x*_MnO_2_ cathode to deliver energy density of ∼230 Wh kg^−1^ (based on the mass of K_*x*_MnO_2_ cathode) at high levels of electrical power and retain ∼100% capacity after a long-term charge/discharge cycling measurement, remarkably outperforming the battery based on monometallic Zn anode. By adjusting the anode-to-cathode mass ratio to 3:1, the overall energy density of Zn-Mn AR-ZIB can reach ∼142 Wh kg^−1^ based on total mass of anode and cathode. The strategy of eutectic-composition alloying can also be extended to other metal anodes for the development of next-generation secondary batteries.

## Methods

### Preparation of Zn-Al alloys and K_*x*_MnO_2_ nanofibers

The Zn_*x*_Al_100−*x*_ (*x* = 50, 88, 100 at%) alloys made of high-purity Zn (99.994%) and Al (99.996%) were prepared by induction melting in high-purity alumina crucibles within Ar air. These alloy ingots were produced through pouring casting, of which the cooling rates were controlled by making use of different casting moulds, i.e., the heated iron moulds (∼10 K s^−1^) and the copper moulds with air- (∼30 K s^−1^) and water-cooling (∼300 K s^−1^) methods. The as-cast Zn_*x*_Al_100−*x*_ ingots were cut into alloy sheets with thickness of ∼400 μm along the perpendicular direction of lamellar structure and further polished for the use as the anodic electrodes. The synthesis of K_0.12_MnO_2_ nanobelts was carried out by a modified hydrothermal method. Typically, the Teflon-lined steel autoclave filled with the mixture of 40-mM KMnO_4_ and 40-mM NH_4_Cl was heated at 150 °C for 24 h in an oil bath and magnetically stirred at a speed of 250 rpm. The as-synthesized K_0.12_MnO_2_ nanomaterials were collected and washed with ultrapure water for five times using a centrifuge to remove residues.

### Structural and chemical characterizations

The metallographic microstructure of Zn_*x*_Al_100−*x*_ alloy sheets was investigated by using a confocal laser scanning microscope (OLS3000, Olympus) after conventional grinding and mechanical polishing, followed by chemical etching in acetic picric solution (5 ml HNO_3_ and 5 ml HF, 90 ml ultrapure water). The electron micrographic structures were characterized by using a field-emission scanning electron microscope (JEOL, JSM-6700F, 15 kV) equipped with an X-ray energy-dispersive microscopy, and a field-emission transmission electron microscope (JEOL, JEM-2100F, 200 kV). XRD measurements were conducted on a D/max2500pc diffractometer using Cu *K*α radiation. Ion concentrations in electrolytes were analyzed by inductively coupled plasma optical emission spectrometer (ICP-OES, Thermo electron). XPS analysis was conducted on a Thermo ECSALAB 250 with an Al anode. Charging effects were compensated by shifting binding energies based on the adventitious C 1s peak (284.8 eV).

### Electrochemical measurements

Symmetrical cells were assembled with two identical Zn_*x*_Al_100-*x*_ alloy or pure Zn sheets (0.5 cm × 0.5 cm × 40 μm), which were separated by glass fiber membrane (GFM) in 2 M ZnSO_4_ aqueous solution with/without N_2_ purgation. Electrochemical stripping/plating behaviors of Zn/Zn^2+^ were measured by galvanostatic charge and discharge at various current densities from 1 to 5 mA cm^−2^. The cycling durability tests were performed at the current density of 0.5 mA cm^−2^. To prove its feasibility of the lamella-structured eutectic Zn_88_Al_12_ alloy anodes in practical aqueous rechargeable Zn-ion batteries, full cells were further assembled with the Zn_88_Al_12_ alloy sheet as the anode, the K_0.12_MnO_2_ as the cathode, the GFM as the separator, with the 2M ZnSO_4_ aqueous solution containing 0.2 M MnSO_4_ as the aqueous electrolyte. Therein, the K_0.12_MnO_2_ electrodes were prepared by homogeneously mixing K_0.12_MnO_2_ nanobelts, super-P acetylene black conducting agent and poly(vinylidene difluoride) binder with a weight ratio of 70:20:10 in N-methyl-2-pyrrolidone (NMP), and then pasting on stainless steel foil with the loading mass of 1.0 mg cm^−2^. Cyclic voltammetry was conducted on an electrochemical analyzer (Ivium Technology) in the voltage range of 1 and 1.8 V at scan rates from 0.3 to 5 mV s^−1^. Electrochemical impedance spectroscopy (EIS) measurements were performed in sealed cells with O_2_- or N_2_-saturated aqueous 2 M ZnSO_4_ electrolytes over the frequency ranging from 100 kHz to 10 mHz with an amplitude of 10 mV at room temperature. The rate capability and cycling performance were carried out on a battery test system. Self-discharge measurements were carried out by charging Zn_88_Al_12_/K_*x*_MnO_2_ to 1.8 V, followed by open-circuit potential self-discharging for 600 h. The coulombic efficiency (CE) of Zn plating/stripping was evaluated by chronocoulometry method, in which the eutectic Zn_88_Al_12_ alloy or pure Zn electrode were used as the working electrode and the Zn foils as the counter and reference electrodes in the three-electrode cell in the O_2_-absent 2 M ZnSO_4_ electrolyte. The chronocoulometry measurements were conducted at the potential of −0.2 and 0.2 V (versus Zn/Zn^2+^) for 600 s, respectively to plate and stripe Zn. The CE was calculated by the stripping/plating capacities.

## Supplementary information

Supplementary Information

Peer Review File

## Data Availability

All relevant data are available from the corresponding authors upon request.
