# Supplementary material for: Lamella-nanostructured eutectic zinc–aluminum alloys as reversible and dendrite-free anodes for aqueous rechargeable batteries
Source: Nat Commun. 2020 Apr 2;11:1634. doi: 10.1038/s41467-020-15478-4 (PMC7118111; doi:10.1038/s41467-020-15478-4)
Supplement: Supplementary file 1 — Supplementary Information [file 41467_2020_15478_MOESM1_ESM.pdf]

Supplementary Information

Lamella-nanostructured eutectic zinc-aluminum alloys as reversible and dendrite-free anodes for aqueous rechargeable batteries

Wang et al

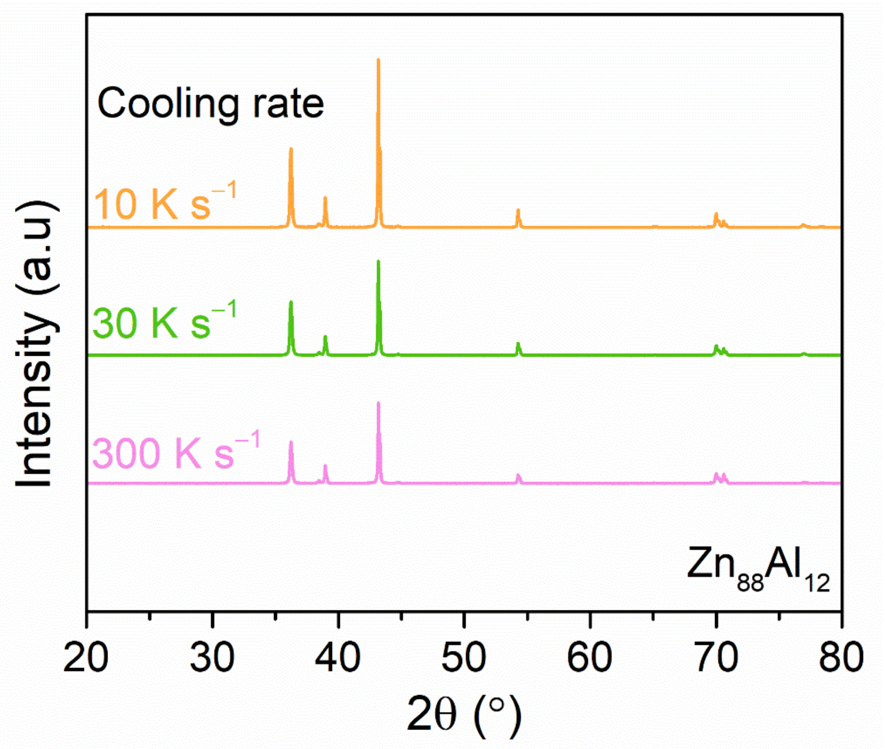

Supplementary Figure 1. XRD patterns of lamella-structured eutectic $Zn_{88}Al_{12}$ alloys that are prepared with different cooling rates from ~10 to ~300 K s^{-1}.

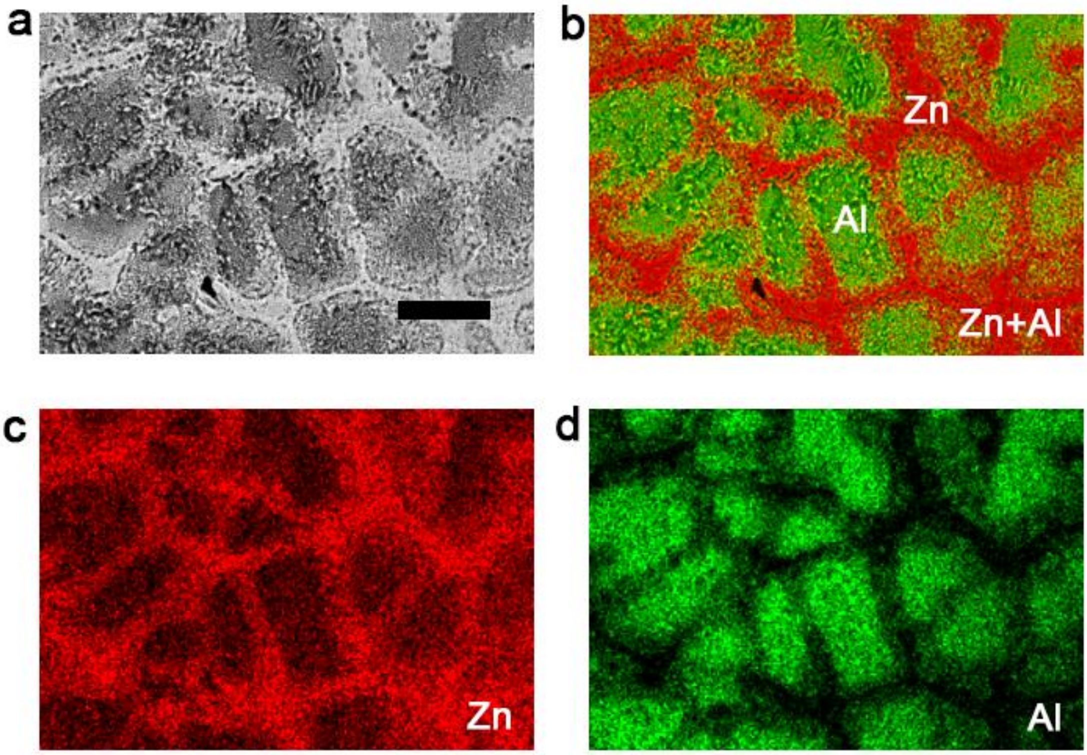

Supplementary Figure 2. Element mapping of hypoeutectic $Zn_{50}Al_{50}$ alloy. a, SEM image of hypoeutectic $Zn_{50}Al_{50}$ alloys. **b**, Mix distribution of Zn and Al. **c**, **d**, Zn (c) and Al (d) element distribution in hypoeutectic $Zn_{50}Al_{50}$ alloy. Scale bar, 10 μm.

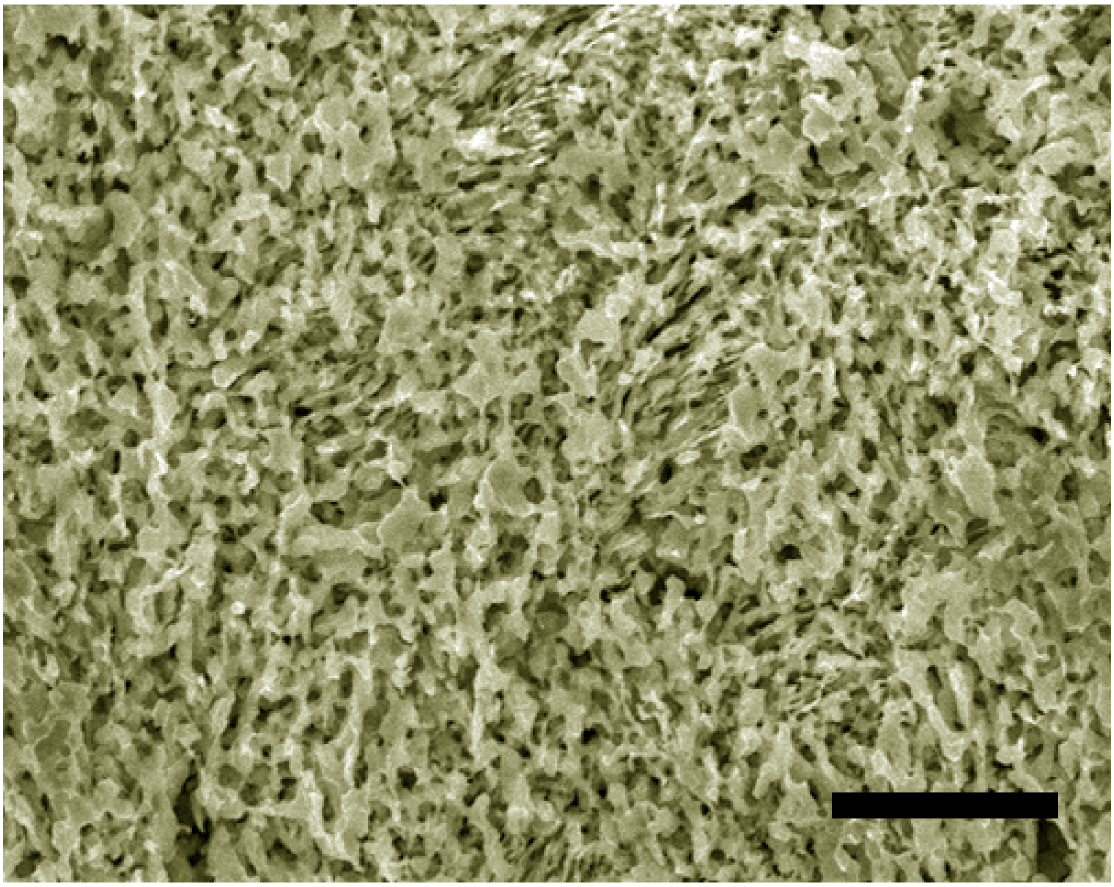

Supplementary Figure 3. Microstructure of etched hypoeutectic $Zn_{50}Al_{50}$ alloy. Typical SEM image of hypoeutectic $Zn_{50}Al_{50}$ alloy after etching in the Keller reagent for 45 s. Scale bar, 5 μm.

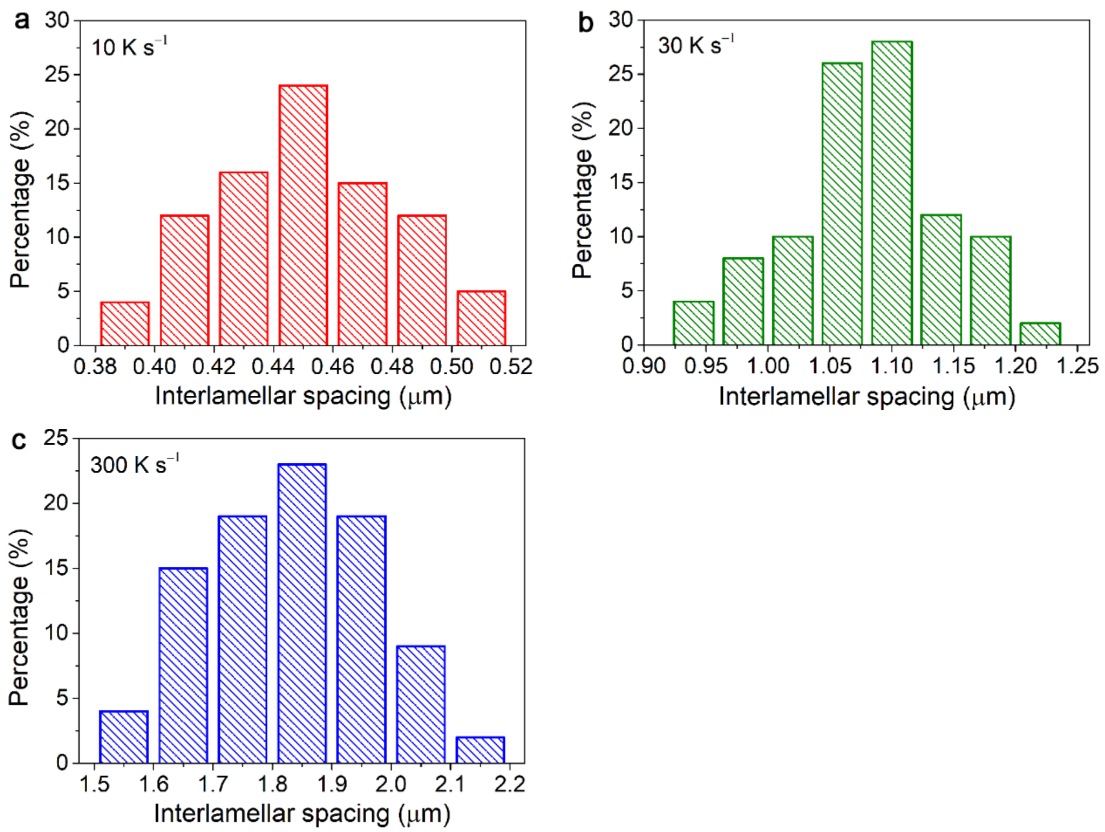

Supplementary Figure 4. Distributions of intelamellar spacing of eutectic $Zn_{88}Al_{12}$ alloys at different cooling rates. a, ~10 K s^{-1}. **b**, ~30 K s^{-1}. **c**, ~300 K s^{-1}.

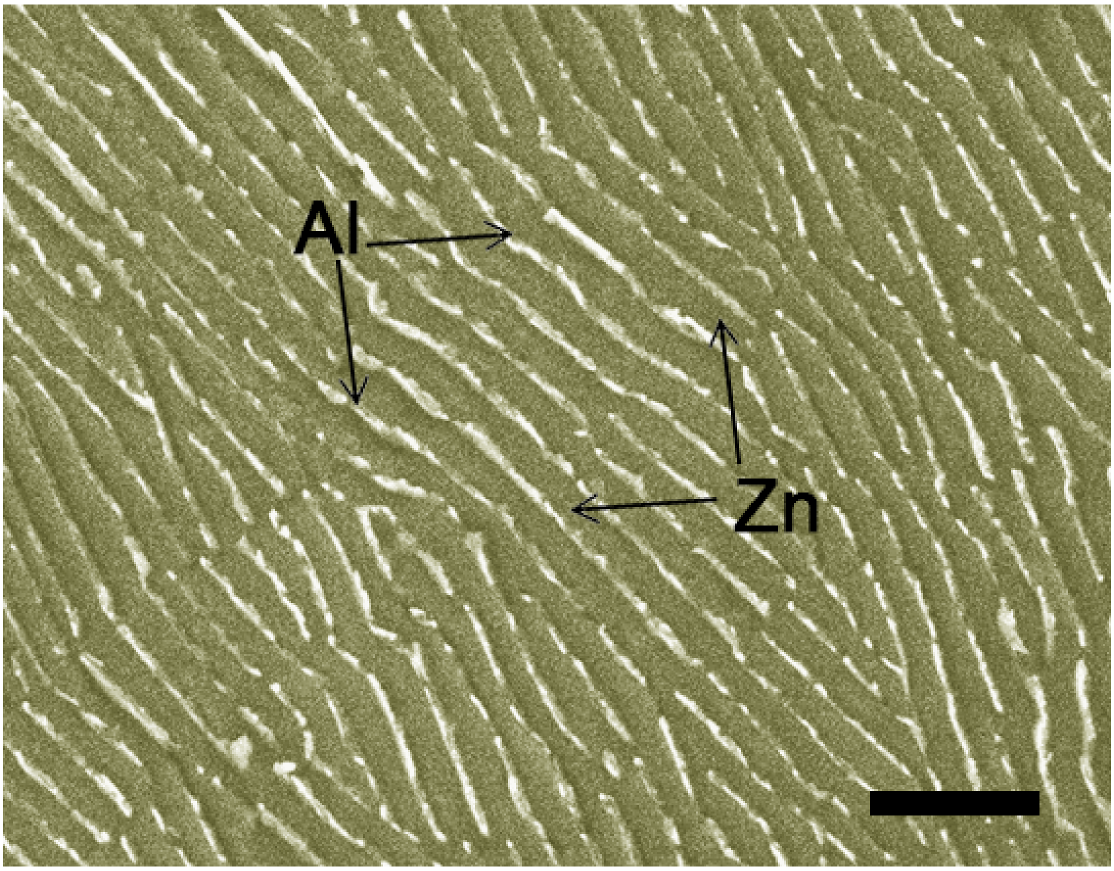

Supplementary Figure 5. Microstructure of etched eutectic $Zn_{88}Al_{12}$ alloy. Typical SEM image of eutectic $Zn_{88}Al_{12}$ alloy with the cooling rate of ~10 K s^{-1} after etching in the Keller reagent for 45 s. Scale bar, 2 μm.

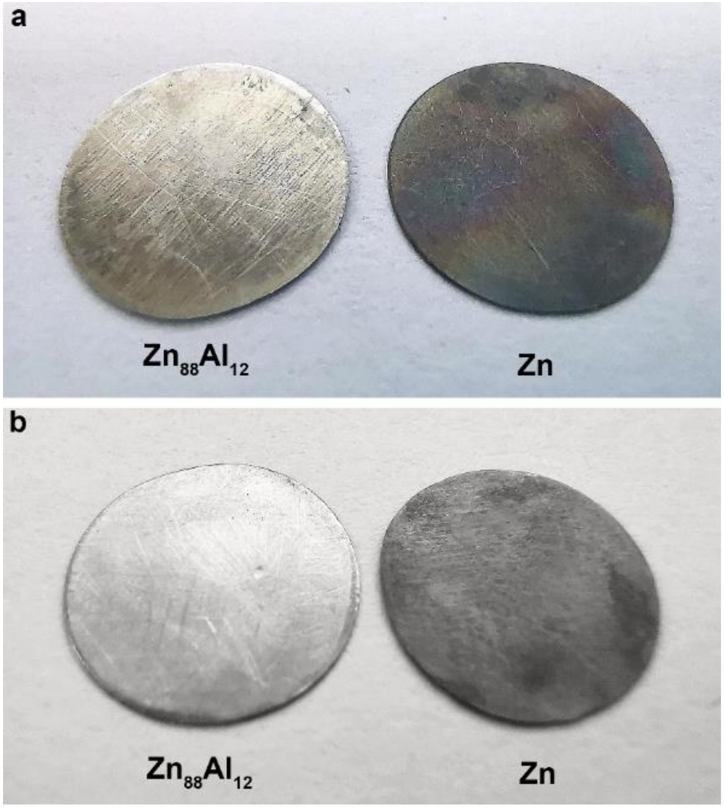

Supplementary Figure 6. Optical photographs comparing $Zn_{88}Al_{12}$ and Zn in air and electrolytes. a, Optical photographs of lamella-structured $Zn_{88}Al_{12}$ alloy with interlamellar spacing of ~450 nm and monometallic Zn in air for five days. **b**, Optical photographs of lamella-structured $Zn_{88}Al_{12}$ alloy with interlamellar spacing of ~450 nm and monometallic Zn in the O_2-present $ZnSO_4$ aqueous electrolyte for 72 hs.

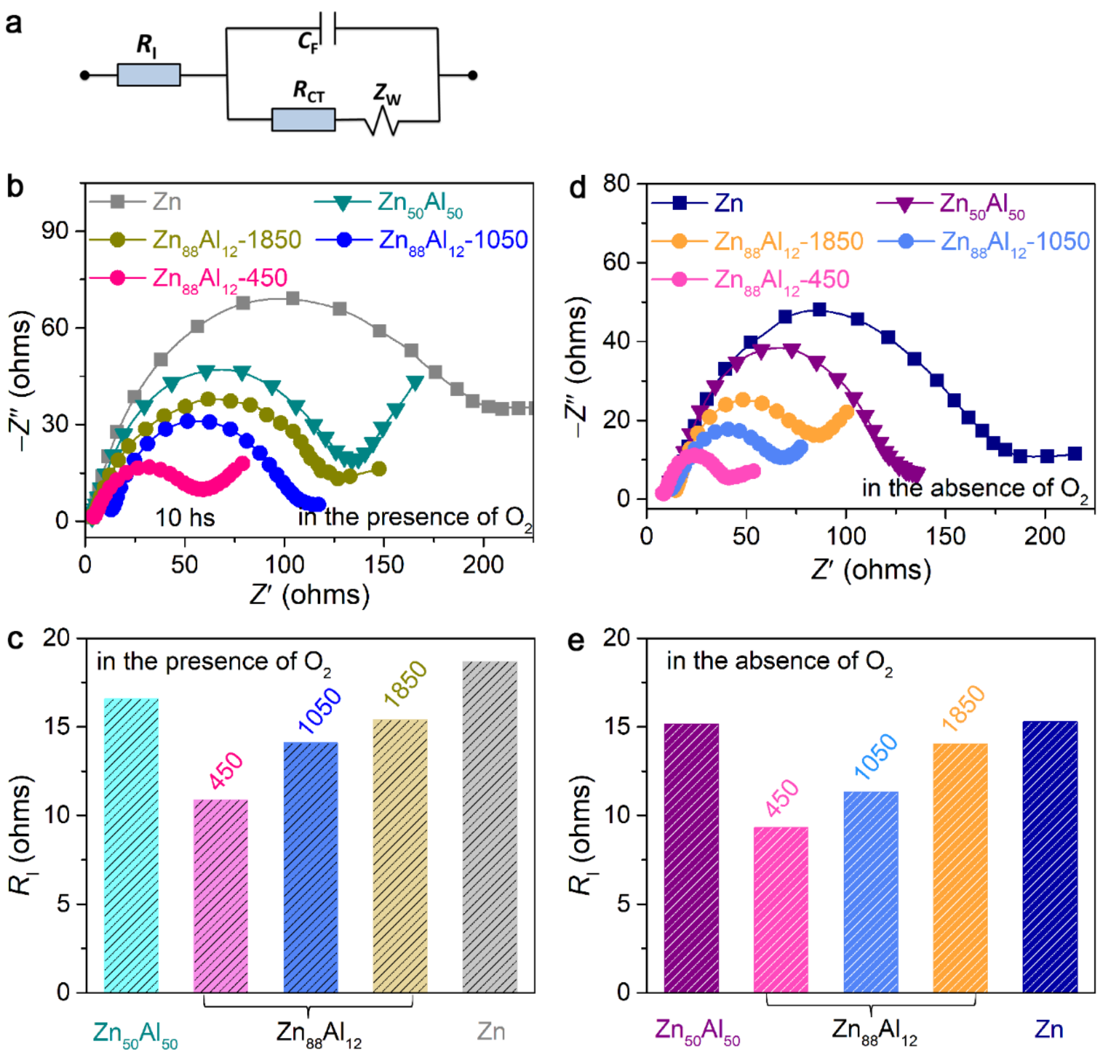

Supplementary Figure 7. EIS and equivalent circuit. a, An equivalent circuit is used to simulate the resistances, where R_I is the ohmic resistance of solution and electrodes, R_{CT} is the charge-transfer resistance, C_F is the double-layer capacitance, and Z_W is the Warburg impedance, respectively. **b**, **d**, EIS spectra of eutectic $Zn_{88}Al_{12}$ alloys with interlamelar spacing of ~450, ~1050 and ~1850 nm, hypoeutectic $Zn_{50}Al_{50}$ alloy and monometallic Zn in the O_2-present (b) or O_2-absent (d) $ZnSO_4$ aqueous electrolyte for 10 h. **c**, Comparison of the R_I values of the eutectic $Zn_{88}Al_{12}$ alloys (~450, ~1050, ~1850 nm), hypoeutectic $Zn_{50}Al_{50}$ alloy and monometallic Zn in the O_2-present aqueous electrolyte for 1 h. **e**, Comparison of the R_I values of the eutectic $Zn_{88}Al_{12}$ alloys (~450, ~1050, ~1850 nm), hypoeutectic $Zn_{50}Al_{50}$ alloy and monometallic Zn in the O_2-absent aqueous electrolyte for 1 h.

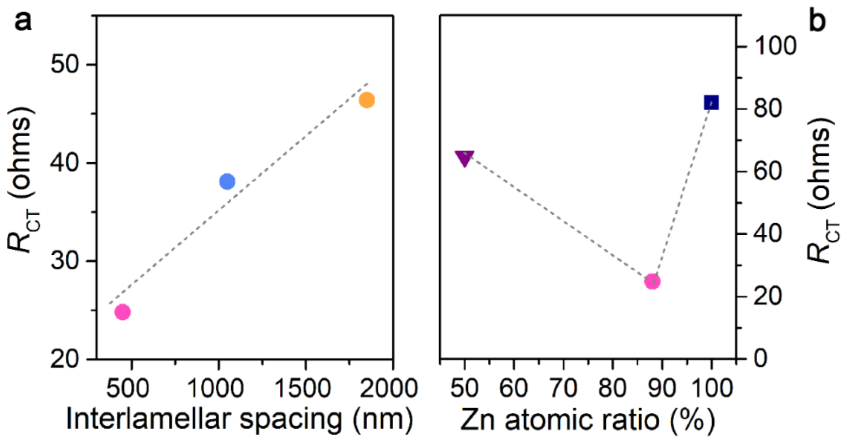

Supplementary Figure 8. Charge transfer resistance (R_{CT}) of Zn and Zn-Al alloys after immersing in aqueous $ZnSO_4$ electrolyte without the presence of O_2 for 10 h. **a**, R_{CT} values of eutectic $Zn_{88}Al_{12}$ alloys with interlamellar spacing of ~450, ~1050 and ~1850 nm, respectively. **b,** R_{CT} values of Zn and Zn-Al alloys as a function of Zn atom ratio.

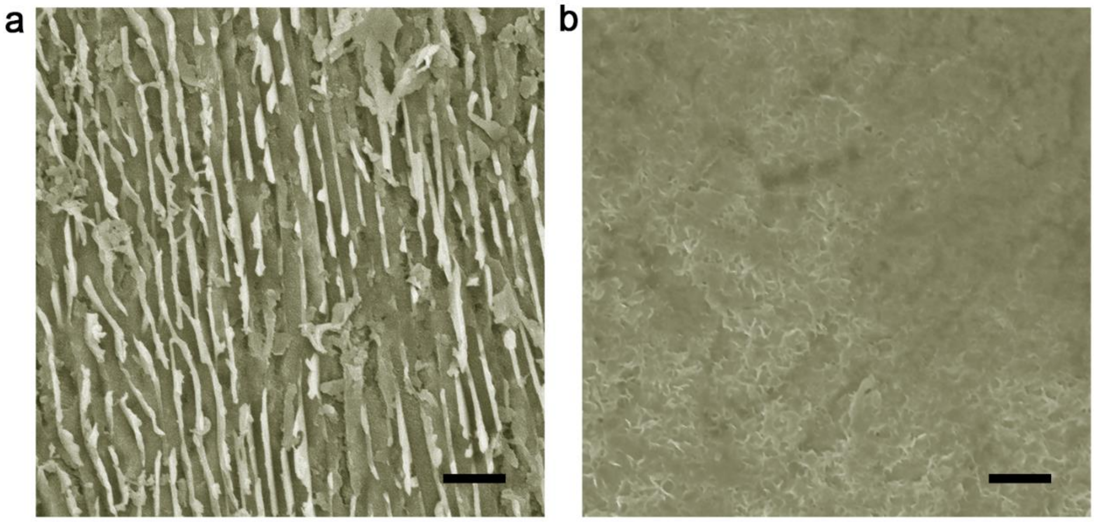

Supplementary Figure 9. Microstructure characterization of eutectic $Zn_{88}Al_{12}$ electrode during the Zn stripping/plating in the O_2-present $ZnSO_4$ aqueous electrolyte. **a**, Typical SEM image of the eutectic $Zn_{88}Al_{12}$ electrode after Zn stripping at the current density of 2 mA cm^{-2} for 10 h. **b**, Representative SEM image of the eutectic $Zn_{88}Al_{12}$ electrode after Zn stripping and then plating at the current density of 2 mA cm^{-2} for 10 h. Scale bar, 1 μm.

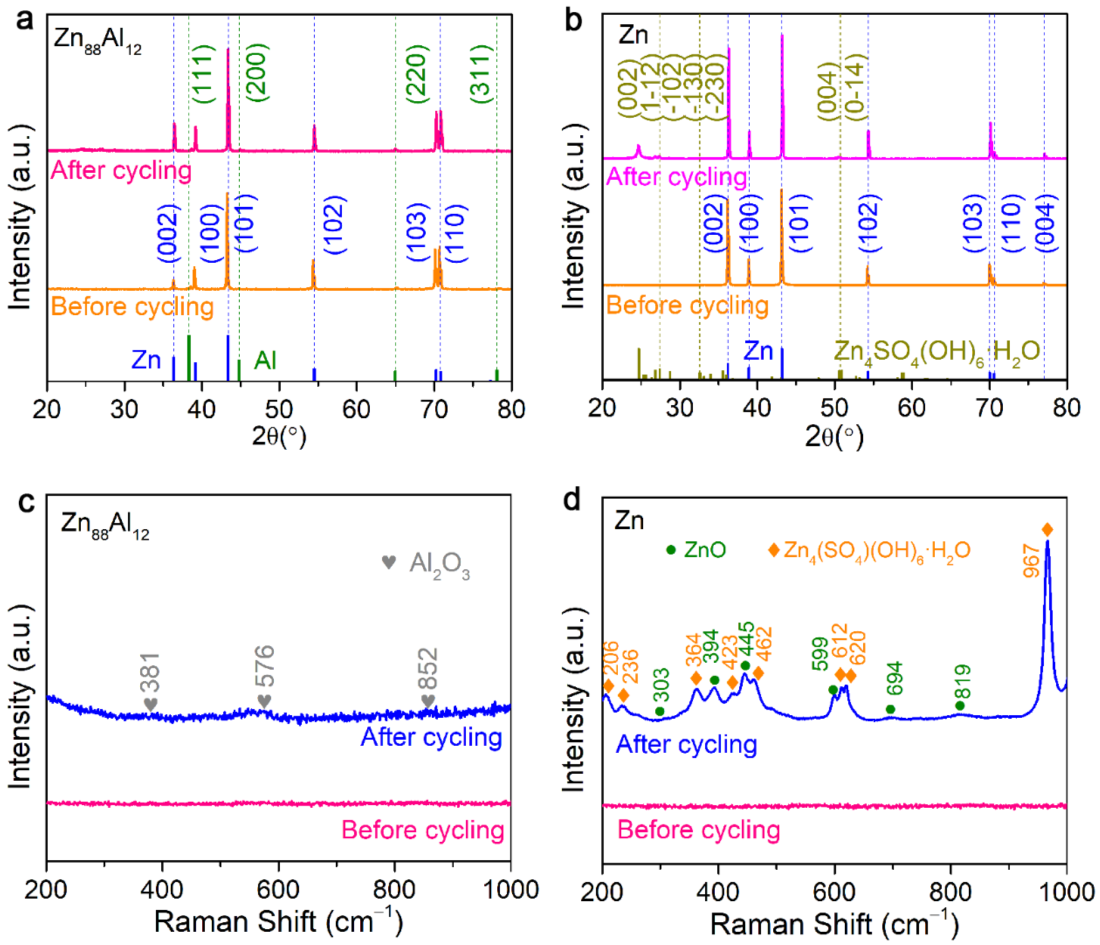

Supplementary Figure 10. XRD and Raman characterizations of eutectic $Zn_{88}Al_{12}$ alloy and monometallic Zn electrode before and after cycling measurements for 20 cycles. a, c, XRD patterns (a) and Raman spectra (c) of the eutectic $Zn_{88}Al_{12}$ alloy before and after cycling measurement in the O_2-absent $ZnSO_4$ aqueous electrolyte. The line patterns show reference cards 04-0831 for hcp Zn (blue) and 04-0787 for fcc Al (gree) according to JCPDS. **b**, **d**, XRD patterns (b) and Raman spectra (d) of the monometallic Zn electrode before and after cycling measurement in the O_2-absent $ZnSO_4$ aqueous electrolyte. The line patterns show reference cards 04-0831 for hcp Zn (blue) and 39-0690 for $Zn_4SO_4(OH)_6{\cdot}H_2O$ (dark yellow) according to JCPDS.

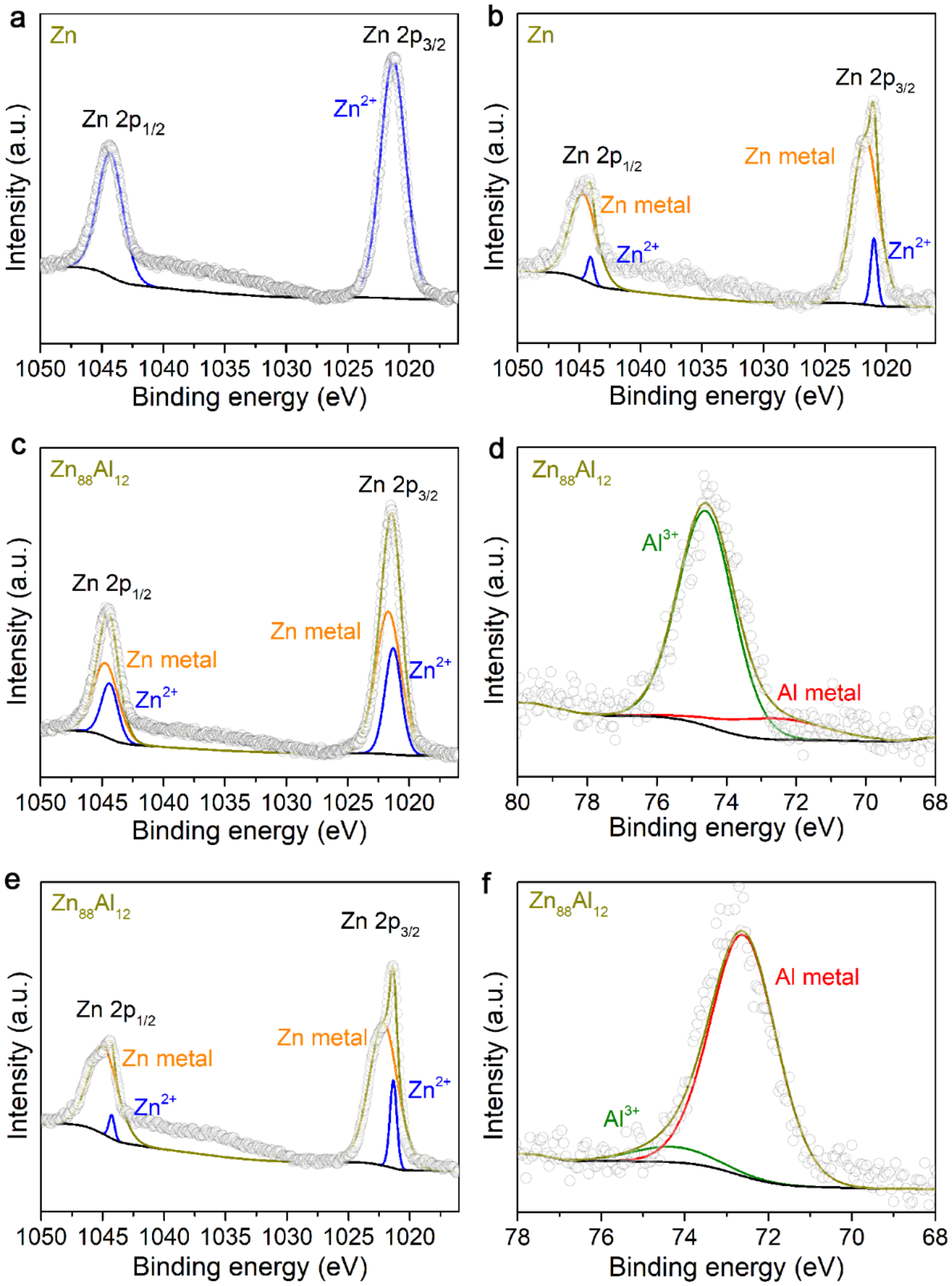

Supplementary Figure 11. XPS characterizations of monometallic Zn and eutectic $Zn_{88}Al_{12}$ alloy before and after cycling measurements for 20 cycles. a, **b**, The Zn 2p XPS spectrum of the monometallic Zn electrode after (a) and before (b) cycling test. **c**, **d**, The Zn 2p (c) and Al 2p (d) XPS spectra of the eutectic $Zn_{88}Al_{12}$ electrode after cycling test. **e**, **f**, The Zn 2p (e) and Al 2p (f) XPS spectra of the eutectic $Zn_{88}Al_{12}$ electrode before cycling test.

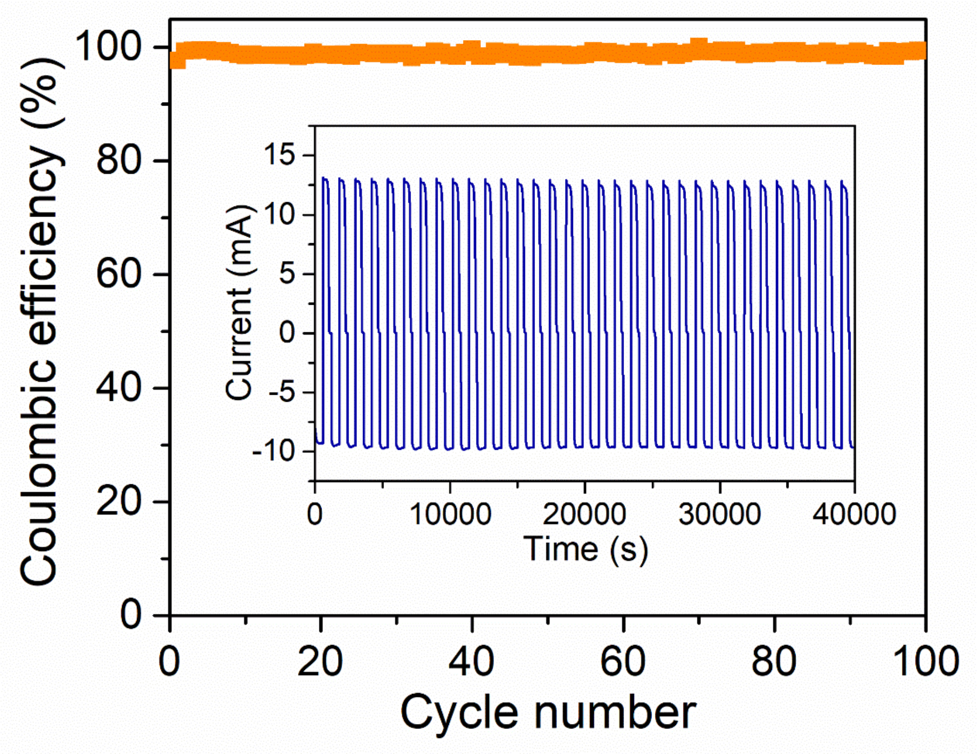

Supplementary Figure 12. The coulombic effect of Zn stripping/plating of the eutectic $Zn_{88}Al_{12}$ alloy with λ = ~450 nm. Inset: chronoamperometry curves at potential of –0.2 and 0.2 V (versus Zn/Zn^{2+}).

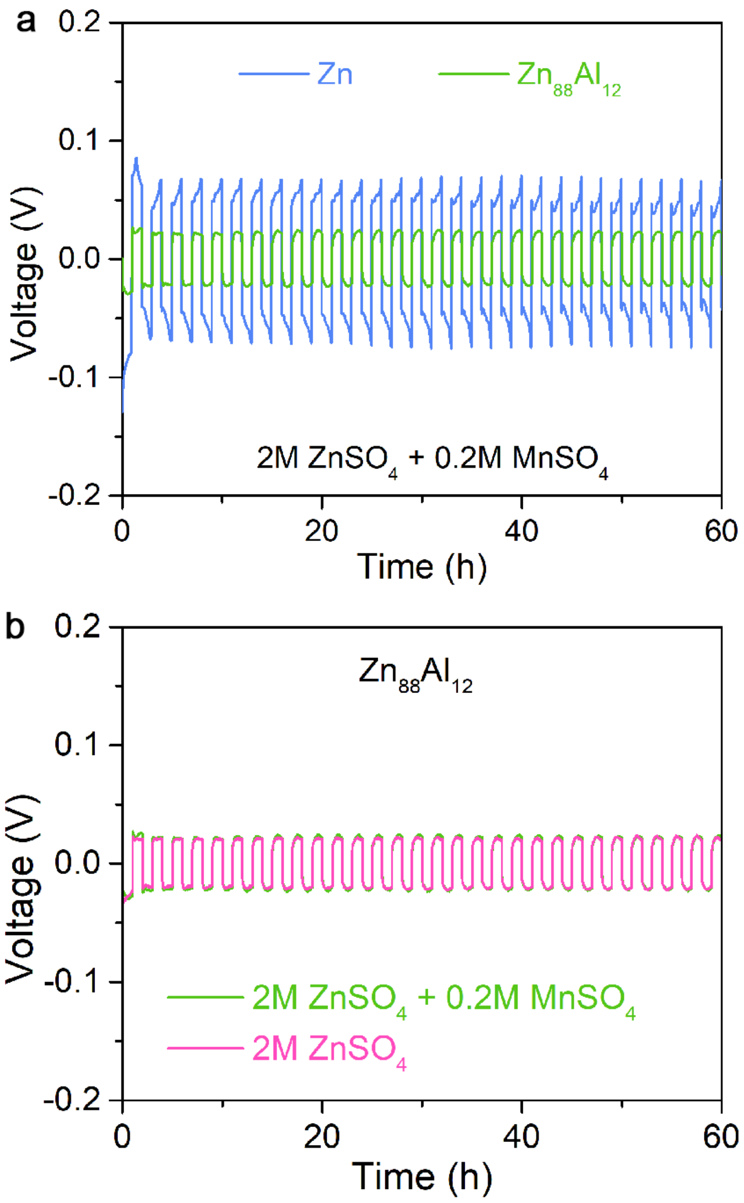

Supplementary Figure 13. Electrochemical characterization in the electrolyte of 2 M $ZnSO_4$ and 0.2 M $MnSO_4$. a, Comparison of voltage profiles for symmetric cells that are constructed with eutectic $Zn_{88}Al_{12}$ or monometallic Zn in the O_2-absent aqueous electrolyte of 2 M $ZnSO_4$ and 0.2 M $MnSO_4$. **b**, Comparison of voltage profile for symmetric cells of the eutectic $Zn_{88}Al_{12}$ alloy in electrolytes of 2 M $ZnSO_4$ with and without 0.2 M $MnSO_4$.

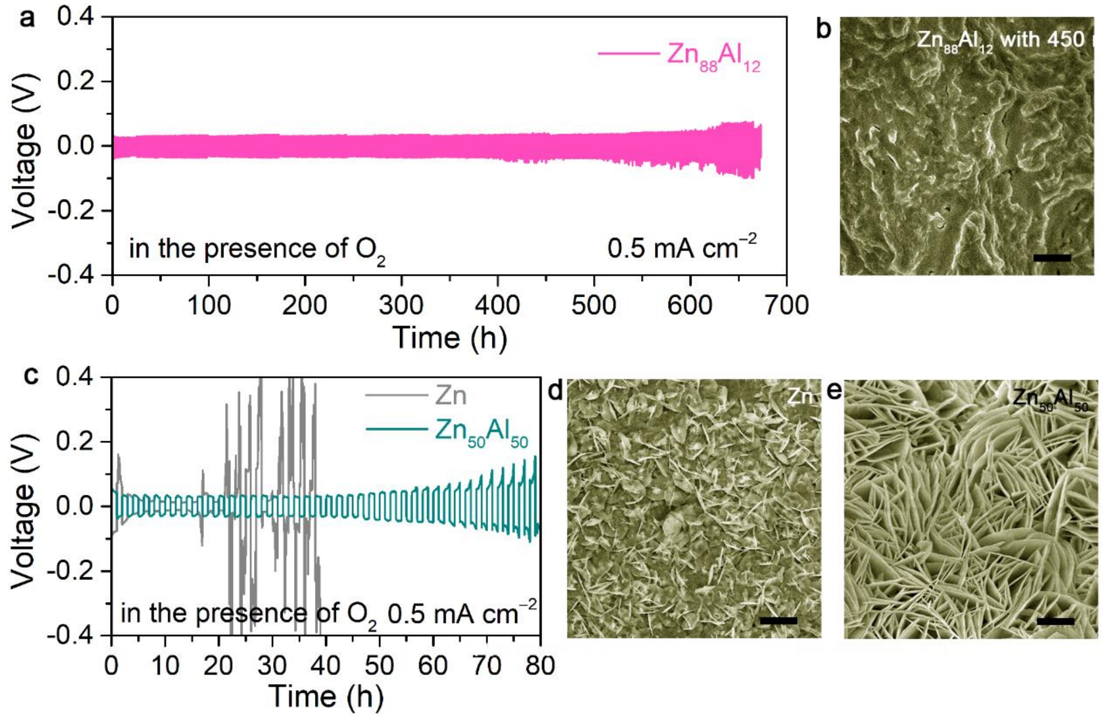

Supplementary Figure 14. Electrochemical performance and microstructure of eutectic $Zn_{88}Al_{12}$, hypoeutectic $Zn_{50}Al_{50}$ and monometallic Zn during the long-term stripping/plating cycling in the O_2-present $ZnSO_4$ aqueous electrolyte. **a**, Voltage profiles for symmetric batteries using eutectic $Zn_{88}Al_{12}$ alloy as electrodes during Zn stripping/plating processes in the O_2-present $ZnSO_4$ aqueous electrolyte at current density of 0.5 mA cm^{-2}. **b**, SEM image of eutectic $Zn_{88}Al_{12}$ alloy after the long-term stripping/plating cycling measurements for 672 hs. Scale bar, 2 μm. **c**, Voltage profiles for symmetric batteries using monometallic Zn or hypoeutectic $Zn_{50}Al_{50}$ alloy as electrodes during Zn stripping/plating processes in the O_2-present $ZnSO_4$ aqueous electrolyte at current density of 0.5 mA cm^{-2}. **d**,**e**, SEM images of monometallic Zn (d), hypoeutectic $Zn_{50}Al_{50}$ alloy(e) after the stripping/plating cycling measurements for 38, 80 hs, respectively. Scale bar, 2 μm.

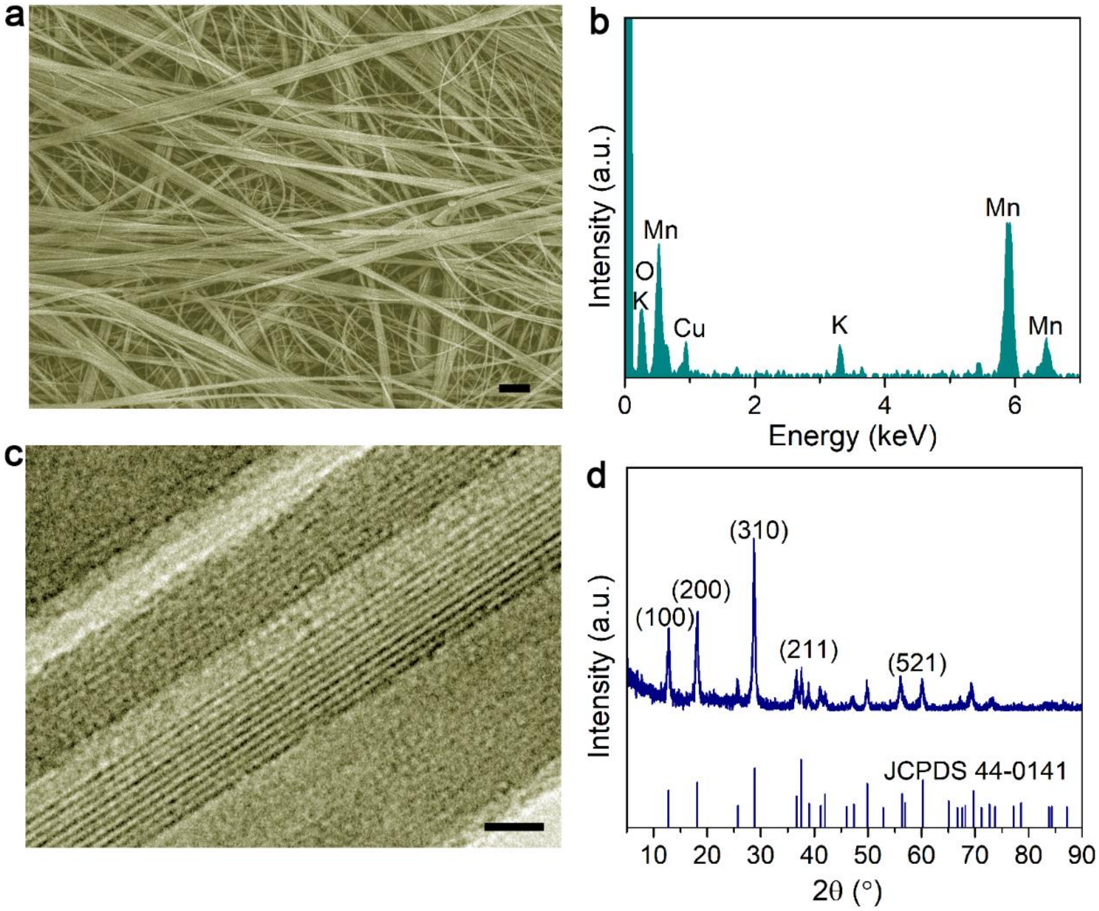

Supplementary Figure 15. Microstructure characterization of cathode of $K_{0.12}MnO_2$ nanofibers. a, Typical SEM image of $K_{0.12}MnO_2$ nanofibers, which are used as cathode of zinc-manganese oxide batteries. Scale bar, 2 μm. **b**, SEM-EDS spectrum of $K_{0.12}MnO_2$ nanofibers. **c**, HRTEM image of $K_{0.12}MnO_2$ nanofibers, demonstrating the layered structure. Scale bar, 5 nm. **d**, XRD patterns of $K_{0.12}MnO_2$ nanofibers. The line patterns show reference card 44-0141 for tetragonal phase of α-MnO_2 according to JCPDS.

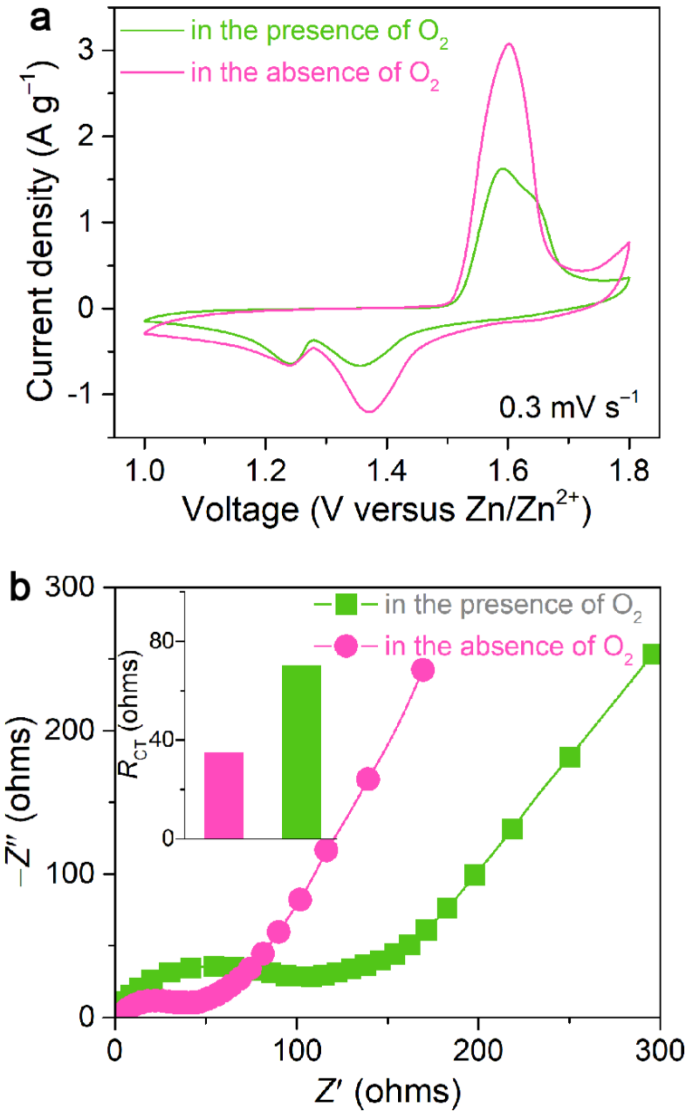

Supplementary Figure 16. Electrochemical properties of $Zn_{88}Al_{12}/K_xMnO_2$ full batteries in aqueous electrolyte with and without the presence of O_2. a, CV curves for full batteries, which are constructed with eutectic $Zn_{88}Al_{12}$ alloy with interlemella spacing of 450 nm as the anode and K_xMnO_2 nanofibers as the cathode ($Zn_{88}Al_{12}$-450/K_xMnO_2), in 2M $ZnSO_4$ aqueous electrolyte with and without the presence of O_2. Scan rate: 0.3 mV s^{-1}. **b**, EIS spectra of $Zn_{88}Al_{12}$-450/K_xMnO_2 full batteries and their corresponding charge transfer resistance (R_{CT}) (inset) in 2M $ZnSO_4$ aqueous electrolyte with and without the presence of O_2.

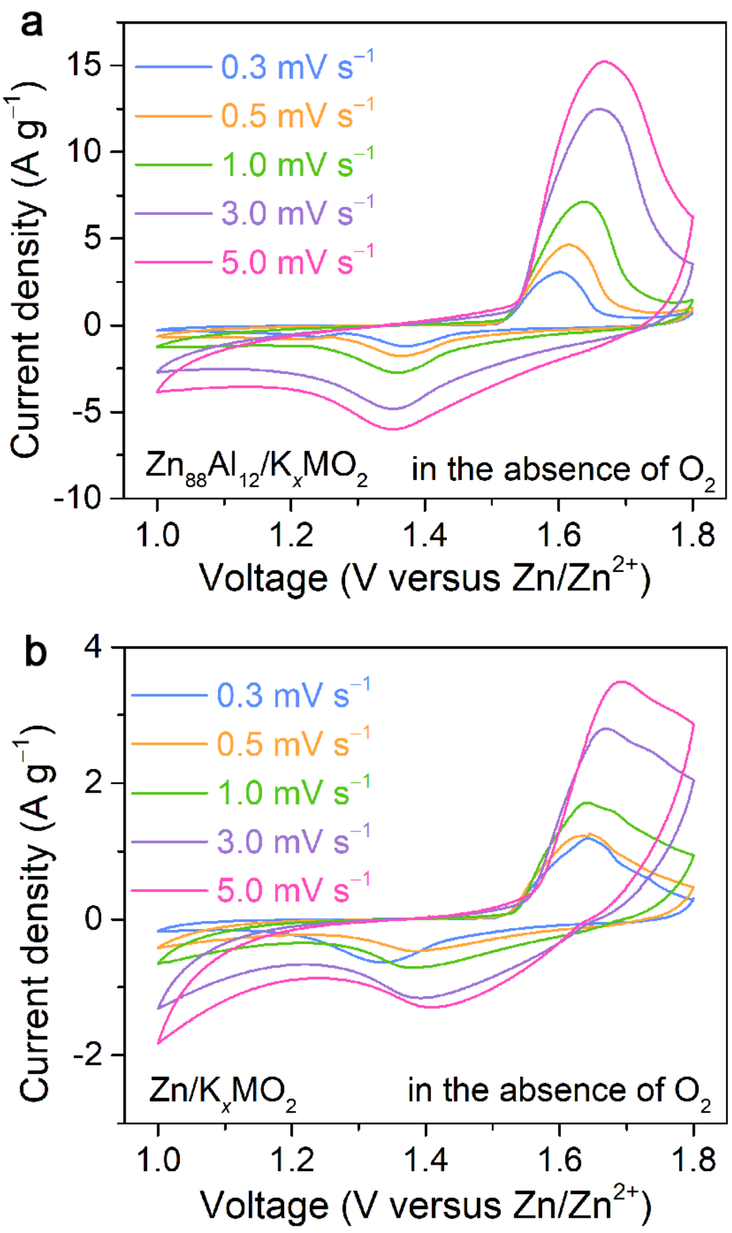

Supplementary Figure 17. Electrochemical properties of $Zn_{88}Al_{12}/K_xMnO_2$ and Zn/K_xMnO_2 full batteries in the O_2-absent aqueous electrolyte. a, CV curves of $Zn_{88}Al_{12}/K_xMnO_2$ full batteries in the O_2-absent $ZnSO_4$ aqueous electrolyte at various scan rates from 0.3 to 5.0 mV s^{-1}. **b**, CV curves of Zn/K_xMnO_2 full batteries in the O_2-absent $ZnSO_4$ aqueous electrolyte at various scan rates from 0.3 to 5.0 mV s^{-1}.

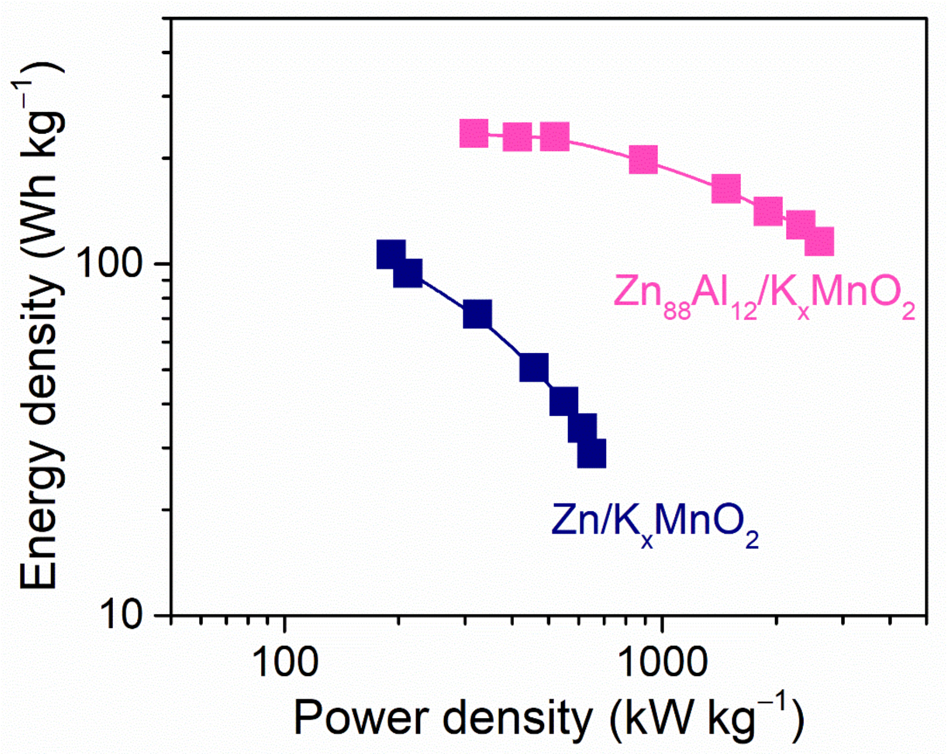

Supplementary Figure 18. Ragone plot comparing power and energy densities of $Zn_{88}Al_{12}/K_{x}MnO_2$ with the values of $Zn/K_{x}MnO_2$.

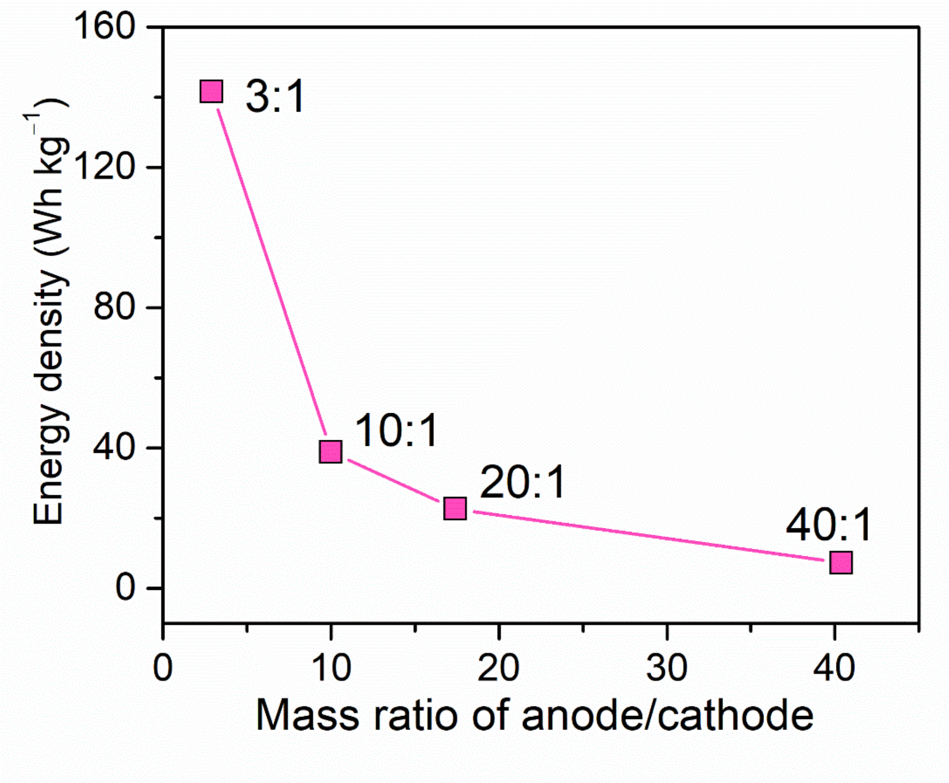

Supplementary Figure 19. Dependence of cell-level energy density on mass ratio of anode and cathode in $Zn_{88}Al_{12}$/K_xMnO_2 full batteries.

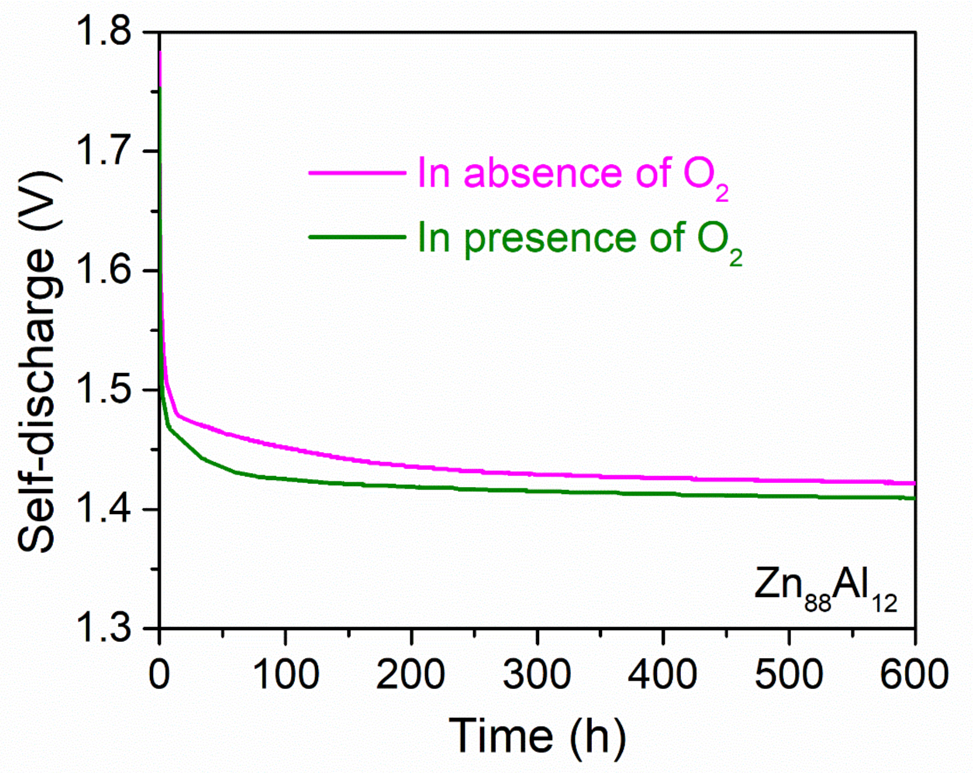

Supplementary Figure 20. Self-discharge performance for $Zn_{88}Al_{12}/K_xMnO_2$ batteries in the 2 M $ZnSO_4$ and 0.2 M $MnSO_4$ electrolytes with/without the presence of O_2.

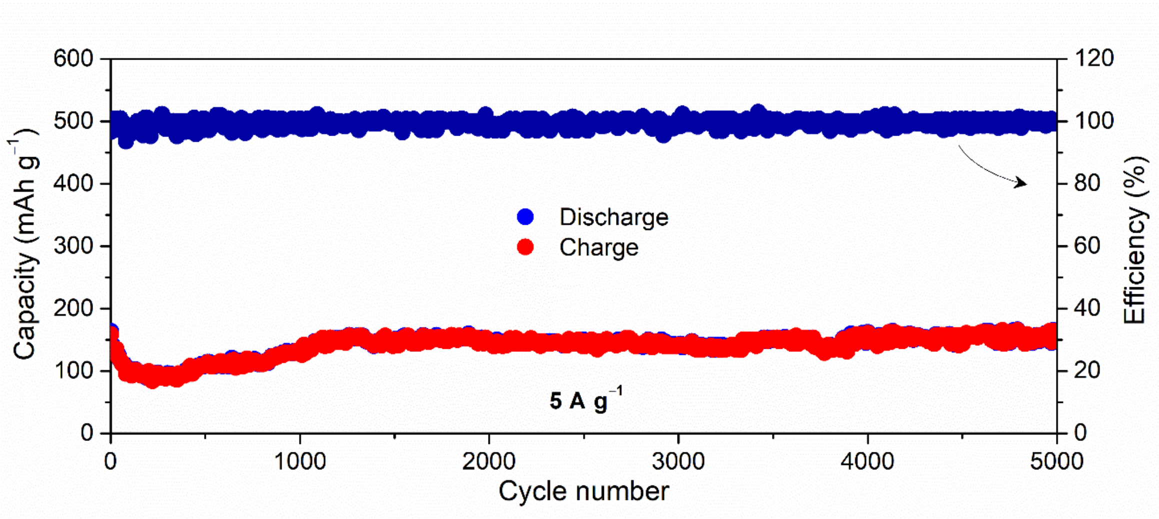

Supplementary Figure 21. Cycling charge/discharge performance of $Zn_{88}Al_{12}/K_xMnO_2$ full battery in the O_2-absent aqueous electrolyte at a current density of 5 A g^{-1}.

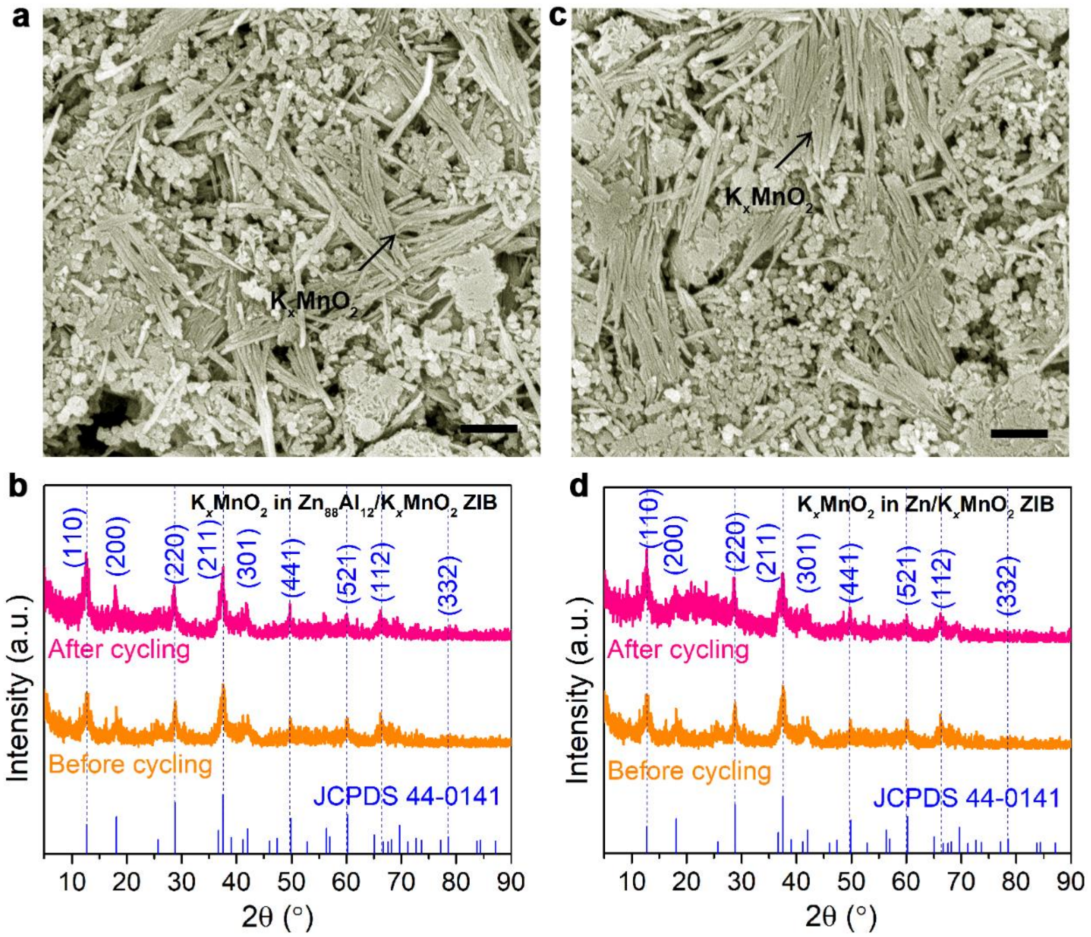

Supplementary Figure 22. Structure characterizations of K_xMnO_2 cathode. a, **b**, Typical SEM image (a) and XRD patterns (b) of K_xMnO_2 cathode of $Zn_{88}Al_{12}/K_xMnO_2$ full battery with the O_2-absent aqueous electrolyte after cycling measurement at 0.5 A g^{-1}. **c**, **d**, Typical SEM image (c) and XRD patterns (d) of K_xMnO_2 cathode of Zn/K_xMnO_2 full battery with the O_2-absent aqueous electrolyte after cycling measurement at 0.5 A g^{-1}. Scale bar, 500 nm (a,c). The line patterns in (b) and (d) show reference card 44-0141 for tetragonal phase of α-MnO_2 according to JCPDS.

Supplementary Table 1. Concentrations of Al^{3+} after electrochemical stripping/plating measurements of symmetric Zn/Zn, $Zn_{50}Al_{50}/Zn_{50}Al_{50}$ and $Zn_{88}Al_{12}/Zn_{88}Al_{12}$ batteries for 300 cycles in 2 M $ZnSO_4$ aqueous electrolyte with and without N_2 purgation.

Specimens	Al^{3+} mass concentrations (mg/mL)		Al^{3+} molar concentrations (mol/L)	
	Electrolyte without O_2	Electrolyte with O_2	Electrolyte without O_2	Electrolyte with O_2
**Zn/Zn**	0	0	0	0
**$Zn_{50}Al_{50}$**	0.0368	1.166	0.0014	0.0417
**$Zn_{88}Al_{12}$-1850**	0	0.922	0	0.0341
**$Zn_{88}Al_{12}$-1050**	0	0.8467	0	0.0314
**$Zn_{88}Al_{12}$-450**	0	0	0	0
