# Supplementary material for: Lamella-nanostructured eutectic zinc–aluminum alloys as reversible and dendrite-free anodes for aqueous rechargeable batteries
Source: Nat Commun. 2020 Apr 2;11:1634. doi: 10.1038/s41467-020-15478-4 (PMC7118111; doi:10.1038/s41467-020-15478-4)
Supplement: Supplementary file 2 — Peer Review File [file 41467_2020_15478_MOESM2_ESM.pdf]

Reviewers' comments:

Reviewer #1 (Remarks to the Author):

An easy alloy strategy was demonstrated in this work to develop highly stable zinc anode and the full zinc-ion battery. The overall idea is based on the prohibition of dendrite formation due to the special surface nanostructure induced by the alloy effect. The electrochemical performance of the full batteries was decent. However, many issues still exist in this paper, which makes it difficult to meet the requirement of Nature Communications:

1. Why the alloy can show this lamellar structure and how it can influence the reversible Zn plating/stripping. Solid evidence and discussions should be provided.

2. The polarization of CD curves of the symmetric batteries using ZnAl alloy was decreased compared to Zn/Zn batteries, though the enhancement was not obvious, and the reason of positive effect from the alloy should be included.

3. Since the mixed solution of $ZnSO_4$ and $MnSO_4$ was used as the electrolyte, more experiments in terms of Zn/Zn batteries should be carried out, and new discussions of the electrolyte effect are needed.

4. The calculation of energy density should also be based on the overall mass of anode and cathode, not just the mass of cathode.

5. What about the self-discharge behavior of the Zn/K_xMnO_2 battery, since both tests in the absence and presence of O_2 had been presented. The self-discharge mechanism should be discussed.

6. In page 9, the authors mentioned that “This observation is probably due to the absence of passivation film on the electroactive Zn surface, which result in the less polarization.” What is the passivation film? XPS data before and after cycling test should be included.

7. Has even the author even tried other metals to form Zn-based alloy (e.g., Li, Mg)? This strategy is potentially a universal way for Zn anodes.

8. There was no capacity fading at 0.5 A g^{-1} in the ZnAl/K_xMnO_2 battery, but the Zn/K_xMnO_2 battery degraded extremely fast. What are the reason besides the effect from the anode? The cathode and electrolyte should be analyzed and discussed for both batteries.

9. The XRD and XPS of the symmetric cells long-term Zn stripping/plating cycling measurements should be provided to see whether there are side reactions on the anodes.

Reviewer #2 (Remarks to the Author):

In this paper, the authors show that eutectic-composition alloying of zinc and aluminum could be an effective strategy to address the irreversibility of Zn anode. The lamellar structure composed of alternating Zn and Al is interesting. The dendrite formation seems to be suppressed in this alloy anode. The discussion of the effect of O_2 in the electrolyte is also instructive. However, some important comments need to be addressed before this paper can achieve the high standard of this reputable journal.

Here are some of my comments:
1. In figure 3, EIS was used to reveal the oxidation resistances of Zn metal and Zn/Al alloys. To be honest, these results are confusing. First of all, the author did not demonstrate how they test the EIS. Are they symmetric cells or not? Did the immersion and oxidation process happen inside a sealed cell or outside in the electrolytes? The author should give the model of the equivalent circuit and calculate the resistance from different components separately. Simply comparing the value does not make sense. And also, charge transfer resistance does not mean the oxidation resistance. Actually, the author should also explain what the oxidation resistance is.
2. In the discussion part of Figure 3 and Figure 4, the author should give more explanation about the reason for the different phenomenon, but not just present the results.
3. When discussing the effect of O2, especially during the electrochemical process in a sealed cell, the author should consider more than just the O2 in the electrolyte. The author could calculate the amount of O2 that dissolved in the electrolyte and compare it with the anode to see whether there is enough O2 to oxidize the alloy. Besides that, the reaction between the alloy and the electrolyte and H2O also need to be taken consideration.
4. The author claimed the high columbic efficiency of the alloy anode; however, no data was demonstrated for the columbic efficiency of the anode.
5. As for the full cell performance, Figure 5b is a little confusing. How did the author get the specific capacity versus the Scan rate?
6. Generally, when the loading of the cathode is only 1.0 mg/cm2, which is much less the anode, the electrochemical performance usually relies on the cathode part but anode part. As in many previous reports, Zn foil can also support a good electrochemical performance. So, in this paper, the author should give more strong and supportive data to demonstrate the superiority of the alloy anode, for example, limiting the anode/cathode mass ratio.

Response To Reviewers′ Comments

Reviewer #1 (Remarks to the Author):

An easy alloy strategy was demonstrated in this work to develop highly stable zinc anode and the full zinc-ion battery. The overall idea is based on the prohibition of dendrite formation due to the special surface nanostructure induced by the alloy effect. The electrochemical performance of the full batteries was decent. However, many issues still exist in this paper, which makes it difficult to meet the requirement of Nature Communications:

Reply: We thank the reviewer for finding interest of our work. We also appreciate the reviewer for his/her constructive comments and suggestions. Following these valuable and insightful comments/suggestions, we have supplemented some experiments, including XRD, Raman and XPS characterizations on the $Zn_{88}Al_{12}$ and Zn electrodes before and after the cycling test of Zn stripping/plating, SEM characterization of $Zn_{88}Al_{12}$ electrode during Zn stripping and then plating, electrochemical characterization on the Zn stripping/plating behavior of $Zn_{88}Al_{12}$ and Zn electrodes in the mixed electrolyte of 2 M $ZnSO_4$ and 0.2 M $MnSO_4$, self-discharge performance of $Zn_{88}Al_{12}/K_xMnO_2$ full batteries, as well as SEM and XRD characterization on the K_xMnO_2 cathodes and ICP-OES measurement of electrolyte in $Zn_{88}Al_{12}/K_xMnO_2$ and Zn/K_xMnO_2 full batteries after cycling measurements. Based on these new results, we have completely revised the manuscript. The detailed corrections are listed below.

(1) Why the alloy can show this lamellar structure and how it can influence the reversible Zn plating/stripping. Solid evidence and discussions should be provided.

Reply: We appreciate the reviewer for the constructive suggestion. As a model system, Zn-Al system is a typical bimetallic alloy with poor miscibility. At the eutectic Zn/Al composition of 88/12 (at %), there takes place a eutectic reaction at the eutectic temperature, in which both Zn and Al constituents crystallize simultaneously from their molten liquid solution. The $Zn_{88}Al_{12}$ alloy often forms a parallel array of the two coexisting phases of Zn and Al lamellas that grow side by side as a result of the balance between the lateral diffusion of excess Zn and Al in the liquid just ahead of the solid/liquid interface and the creation of Zn/Al interfacial area during the solidification process. The eutectic structure composed of alternating Zn and Al lamellas enlists the $Zn_{88}Al_{12}$ alloy electrode to effectively circumvent the irreversibility issues of monometallic Zn anode for potential applications in rechargeable Zn-based batteries. Therein, the Zn lamellas supply the Zn^{2+} charge carriers with the Zn stripping and the residual Al lamellas with self-grown Al_2O_3

shells form lamellar nanopatterns. In view that the insulating Al_2O_3 shells substantially block the electron transfer from Al to the Zn^{2+} cations, there forms a positively electrostatic shield around the Al/Al_2O_3 lamellas to guide the uniform Zn deposition at their interlamellar spacing along the Zn precursor sites. To further demonstrate these processes, we have carried out supplementary SEM characterization on the eutectic $Zn_{88}Al_{12}$ electrode after the Zn stripping and then plating. As shown in supplementary **Figure 9a**, the Zn-stripped $Zn_{88}Al_{12}$ electrode displays evidently lamellar nanopatterns of the residual Al/Al_2O_3 lamellas. While the Zn-stripped $Zn_{88}Al_{12}$ recovers to the initial morphology with a smooth surface as a result of the Al/Al_2O_3 lamellas guiding the subsequent Zn electrodeposition in the plating process (supplementary **Figure 9b**).

(2) The polarization of CD curves of the symmetric batteries using ZnAl alloy was decreased compared to Zn/Zn batteries, though the enhancement was not obvious, and the reason of positive effect from the alloy should be included.

Reply: We thank the reviewer for the comment. Following the comment, we have explained the positive effect of alloy in the text. The less polarization of $Zn_{88}Al_{12}$ is probably due to the unique eutectic structure of alternating Zn and Al lamellas. Therein, the constituent Al lamellas not only protect against the passivation of the electroactive Zn but reduce the local current density of Zn stripping/plating via the formation of core/shell Al/Al_2O_3 lamellar nanopatterns (supplementary **Figure 9a**), which in turn guide the uniform Zn electrodeposition in the subsequent plating process (supplementary **Figure 9b**). The XRD and Raman spectroscopy characterizations evidence the absence of passivation film on the electroactive Zn lamellas of $Zn_{88}Al_{12}$ (supplementary **Figure 10a,c**), which usually forms on the monometallic Zn electrode. As shown in supplementary **Figure 10b,d** for the XRD patterns and the Raman spectrum of the cycled Zn electrode, there appear neoformative diffraction peaks and characteristic Raman bands that correspond to $Zn_4SO_4(OH)_6{\cdot}H_2O$ in addition to the ZnO. As a result, the eutectic $Zn_{88}Al_{12}$ alloy electrode exhibits a less polarization than the monometallic Zn.

(3) Since the mixed solution of $ZnSO_4$ and $MnSO_4$ was used as the electrolyte, more experiments in terms of Zn/Zn batteries should be carried out, and new discussions of the electrolyte effect are needed.

Reply: We thank the reviewer for this constructive suggestion. Following this suggestion, we have performed additional measurements of Zn stripping/plating on the basis of symmetric cells constructed with identical $Zn_{88}Al_{12}$ or Zn electrodes in the electrolyte containing 2 M $ZnSO_4$ and 0.2 M $MnSO_4$. As shown in supplementary

Figure 13, the lamella-structured $Zn_{88}Al_{12}$ alloy electrode exhibits almost the same voltage profile in the mixed solution of 2 M $ZnSO_4$ and 0.2 M $MnSO_4$ as in the 2 M $ZnSO_4$ aqueous solution. It demonstrates that the addition of 0.2 $MnSO_4$ does not influence the electrochemical behavior of $Zn_{88}Al_{12}$ alloy electrode.

(4) The calculation of energy density should also be based on the overall mass of anode and cathode, not just the mass of cathode.

Reply: Following this comment, we have calculated the energy density on the basis of the total mass of anode and cathode. The overall energy density of the full $Zn_{88}Al_{12}/K_xMnO_2$ battery can reach ~142 Wh kg^{-1} by lowering the anode-to-cathode mass ratio to 3:1 (supplementary **Figure 19**).

(5) What about the self-discharge behavior of the Zn/KxMnO$_2$ battery, since both tests in the absence and presence of O$_2$ had been presented. The self-discharge mechanism should be discussed.

Reply: We appreciate the reviewer for the suggestion, according to which we have performed self-discharge measurement of the $Zn_{88}Al_{12}/K_xMnO_2$ battery in the 2 M $ZnSO_4$ and 0.2 M $MnSO_4$ electrolytes with/without the presence of O_2. Supplementary **Figure 20** shows the self-discharge performance of the $Zn_{88}Al_{12}/KxMnO_2$ batteries. In the electrolyte with the absence of O_2, the voltage dramatically drops to ~1.481 V from 1.800 V in ~13 h. This is slower than the $Zn_{88}Al_{12}/K_xMnO_2$ battery with the O_2-present electrolyte, of which the voltage decreases to ~1.472 V in ~6 h. The initial voltage drop is probably due to the pseudocapacitive discharge behavior of K_xMnO_2, which is boosted by the presence of O_2. Nevertheless, the $Zn_{88}Al_{12}/K_xMnO_2$ batteries in the electrolyte with/without the presence of O_2 exhibit a voltage plateau with very low self-discharge, with ~0.1 mV h^{-1}, in the subsequent 600 h, because of ultralow insertion kinetics of Zn^{2+}, which is almost independent of O_2 in the electrolyte.

(6) In page 9, the authors mentioned that "This observation is probably due to the absence of passivation film on the electroactive Zn surface, which result in the less polarization." What is the passivation film? XPS data before and after cycling test should be included.

Reply: We thank the reviewer for the constructive comment and suggestion, according to which we have carried out supplementary XRD, Raman and XPS characterizations on the $Zn_{88}Al_{12}$ and Zn electrodes before and after cycling test. The detailed results are shown in supplementary **Figure 10 and Figure 11**. After cycling test, the monometallic Zn electrode displays neoformative peaks in both XRD patterns

and Raman spectrum compared with the one before cycling test (supplementary **Figure 10b,d**). These neoformative diffraction peaks are in agreement with the line patterns of $Zn_4SO_4(OH)_6{\cdot}H_2O$ (JCPDS 39-0690), demonstrating the formation of $Zn_4SO_4(OH)_6{\cdot}H_2O$ on the Zn metal (supplementary **Figure 10b**). Raman spectrum of the tested Zn electrode reveals that in addition to the $Zn_4SO_4(OH)_6{\cdot}H_2O$, there is ZnO to form on the surface during the cycling test (supplementary **Figure 10d**). In contrast, the $Zn_{88}Al_{12}$ alloy electrode after cycling test exhibits almost the same XRD patterns as the one before cycling test (supplementary **Figure 10a**). Furthermore, the tested $Zn_{88}Al_{12}$ electrode does not display additional characteristic Raman bands in addition to the weak ones that is due to the Al_2O_3 (supplementary **Figure 10c**). These facts confirm the absence of passivation film on the $Zn_{88}Al_{12}$ electrode, which results in the less polarization. The XPS spectra of the monometallic Zn and the eutectic $Zn_{88}Al_{12}$ alloy before and after cycling test are shown in supplementary **Figure 11**. The Zn 2p XPS demonstrates that the surface Zn on the pristine monometallic Zn electrode is mainly in metallic state in addition to a little oxidized state due to the general surface oxidation (supplementary **Figure 11b**). While for the Zn electrode after cycling test, the surface Zn is in completely oxidized state because of the formation of $Zn_4SO_4(OH)_6{\cdot}H_2O$ and ZnO (supplementary **Figure 11a**). Supplementary **Figure 11c,e** show the Zn 2p XPS spectra of the eutectic $Zn_{88}Al_{12}$ alloy after and before the cycling test, displaying almost the same chemical states of Zn, mainly in metallic Zn in addition to some Zn^{2+}. After the cycling test, the metallic Al primarily becomes Al^{3+} as a result of the formation of Al_2O_3 shell (supplementary **Figure 11d,f**).

(7) Has even the author even tried other metals to form Zn-based alloy (e.g., Li, Mg)? This strategy is potentially a universal way for Zn anodes.

Reply: We appreciate the reviewer for approving our eutectic strategy to be potentially a universal way for Zn anodes. In view that both Zn-Li and Zn-Mg alloy systems have similar eutectic structures, they are expected to hold a promise to circumvent the dendrite problem of Zn anode.

(8) There was no capacity fading at 0.5 A g^{-1} in the ZnAl/KxMnO$_2$ battery, but the Zn/K_xMnO_2 battery degraded extremely fast. What are the reason besides the effect from the anode? The cathode and electrolyte should be analyzed and discussed for both batteries.

Reply: We appreciate the reviewer for the valuable comment and suggestion. Following this suggestion, we have carried out SEM and XRD characterizations on the K_xMnO_2 cathodes in the $Zn_{88}Al_{12}/K_xMnO_2$ and Zn/K_xMnO_2 full batteries after cycling test. Supplementary **Figure 22a,c** show the typical SEM images of the

K_xMnO_2 cathodes in the $Zn_{88}Al_{12}/K_xMnO_2$ and Zn/K_xMnO_2 full batteries, displaying the same morphology. Furthermore, these tested K_xMnO_2 cathodes exhibit the same XRD patterns as the as-prepared one (supplementary **Figure 22b,d**). In addition, ICP-OES analysis demonstrates that the tested electrolyte keeps almost the same concentration of Zn^{2+} and Mn^{2+} ions as the initial one. These facts indicate that the fast capacity degradation of Zn/K_xMnO_2 battery at 0.5 A g^{-1} results from the monometallic Zn anode, which undergoes severe irreversibility issues associated with the dendrite formation and growth and side reaction of $Zn_4SO_4(OH)_6{\cdot}H_2O$ and ZnO (supplementary **Figure 10b,d** and **Figure 11a**).

(9) The XRD and XPS of the symmetric cells long-term Zn stripping/plating cycling measurements should be provided to see whether there are side reactions on the anodes.

Reply: We thank the reviewer for the constructive suggestion, following which we have carried out supplementary XRD and XPS characterizations on the symmetric cells of $Zn_{88}Al_{12}$ and Zn electrodes after the cycling measurement of Zn stripping/plating. At the same time, we also carried out Raman spectroscopy measurements. The detailed results are shown in supplementary **Figure 10** and **Figure 11**. The $Zn_{88}Al_{12}$ electrode displays almost the same XRD patterns before and after the cycling test of Zn stripping/plating, demonstrating that there does not occur evident side reactions on the $Zn_{88}Al_{12}$ alloy, in addition to the formation of Al_2O_3 on the Al lamellar nanopatterns (supplementary **Figure 10c**). While for the monometallic Zn electrode, there occurs evident side reactions to form $Zn_4SO_4(OH)_6{\cdot}H_2O$ and ZnO. As shown in supplementary **Figure 10b,d** for the XRD patterns and the Raman spectrum of the cycled Zn electrode, there appear neoformative diffraction peaks and characteristic Raman bands that correspond to $Zn_4SO_4(OH)_6{\cdot}H_2O$ in addition to the ZnO. As a consequence, X-ray photoelectron spectroscopy (XPS) analysis demonstrates the complete Zn^{2+} state of surface Zn in the Zn electrode (supplementary **Figure 11a**). While the Zn 2p XPS of the $Zn_{88}Al_{12}$ reveals that the surface Zn maintains almost the same chemical states, i.e., in both metallic Zn and Zn^{2+}, as the one before cycling test (supplementary **Figure 11c,e**). the Al primarily becomes Al^{3+} as a result of the formation of Al_2O_3 shell (supplementary **Figure 11d,f**).

Reviewer #2 (Remarks to the Author):

In this paper, the authors show that eutectic-composition alloying of zinc and aluminum could be an effective strategy to address the irreversibility of Zn anode. The lamellar structure composed of alternating Zn and Al is interesting. The dendrite formation seems to be suppressed in this alloy anode. The discussion of the effect of O_2 in the electrolyte is also instructive. However, some important comments need to be addressed before this paper can achieve the high standard of this reputable journal. Here are some of my comments:

Reply: We appreciate the reviewer for his/her insightful and constructive comments and finding our work of interest. According to these comments/suggestions, we have carried out supplementary experiments, including the determination of the amount of O_2 in electrolyte, the electrochemical measurements to demonstrate the coulumbic efficiency of $Zn_{88}Al_{12}$ alloy electrode, as well as the overall energy density of the full $Zn_{88}Al_{12}/K_xMnO_2$ batteries with various anode-to-cathode mass ratios. Based on these new results and the reviewer′s valuable suggestions, we have completely revised the manuscript. The detailed corrections are listed below.

(1) In figure 3, EIS was used to reveal the oxidation resistances of Zn metal and Zn/Al alloys. To be honest, these results are confusing. First of all, the author did not demonstrate how they test the EIS. Are they symmetric cells or not? Did the immersion and oxidation process happen inside a sealed cell or outside in the electrolytes? The author should give the model of the equivalent circuit and calculate the resistance from different components separately. Simply comparing the value does not make sense. And also, charge transfer resistance does not mean the oxidation resistance. Actually, the author should also explain what the oxidation resistance is.

Reply: We appreciate the reviewer for the insightful comments. Following the comments, we have presented a detailed method to perform the EIS measurement in Result and Method sections. We also have corrected the presentation to clearly explain the oxidation-resistance capability of the eutectic $Zn_{88}Al_{12}$ alloy electrode. Our EIS measurements were performed in sealed cells with O_2- or N_2-saturated aqueous 2 M $ZnSO_4$ electrolytes at room temperature, respectively, on the basis of a classic three-electrode configuration with Pt foil as the counter electrode and an Ag/AgCl electrode as the reference electrode. The frequency ranges from 100 kHz to 10 mHz and the amplitude is 10 mV. Because of the high chemical activity of Zn and Al, there always exists an oxide layer on the electrode surface, as demonstrated by XPS analysis in supplementary **Figure 11b,e,f**. Nevertheless, the $Zn_{88}Al_{12}$ alloys exhibit superior oxidation-resistance capability in aqueous electrolyte compared with

the monometallic Zn electrode. This is demonstrated by EIS analysis in **Figure 3a** and supplementary **Figure 7b**, which compare the EIS spectra of $Zn_{88}Al_{12}$ alloys with those of hypoeutectic $Zn_{50}Al_{50}$ alloy and monometallic Zn in the O_2-present electrolytes for 1 and 10 h, respectively. Based on the equivalent circuit with the general descriptors: the intrinsic resistance of solution and electrodes (R_I), the charge-transfer resistance (R_{CT}), the double-layer capacitance (C_F) and the Warburg impedance (Z_W) (supplementary **Figure 7a**), the EIS spectra are analyzed using the complex nonlinear least-squares fitting method. Supplementary **Figure 7c** compares the R_I values of all Zn-based electrodes immersed in the O_2-present electrolyte for 1 h, wherein the $Zn_{88}Al_{12}$ with λ = ~450 nm has the lowest R_I value because of the outstanding oxidation-resistance property. Even extending the immersion time to 10 h, the $Zn_{88}Al_{12}$ with λ = ~450 nm still maintains ~11 Ω whereas the Zn electrode has the R_I value to increase to ~22 Ω. The large change of R_I value indicates the inferior oxidation-resistance capability of monometallic Zn. Owing to their different oxidation-resistance properties, there form distinct oxide layers to depress the Zn stripping/plating kinetics, indicated by the increase of R_{CT} value. When immersed in the O_2-present electrolyte for 1 and 10 h, the $Zn_{88}Al_{12}$ with interlamellar spacing of ~450 nm exhibit exceptional stability with the R_{CT} value changing from ~32 Ω to ~36 Ω, in sharp contrast with the monometallic Zn electrode with a remarkable change of R_{CT} from ~96 Ω to ~177 Ω (**Figure 3b**). These observations demonstrate that the eutectic $Zn_{88}Al_{12}$ alloy electrode exhibit superior oxidation-resistance capability relative to the monometallic Zn electrode.

(2) In the discussion part of Figure 3 and Figure 4, the author should give more explanation about the reason for the different phenomenon, but not just present the results.

Reply: We appreciate the reviewer for the constructive suggestion. According to this suggestion, we have presented more discussion on the EIS results and electrochemical performance. The details are included in the text.

(3) When discussing the effect of O_2, especially during the electrochemical process in a sealed cell, the author should consider more than just the O_2 in the electrolyte. The author could calculate the amount of O_2 that dissolved in the electrolyte and compare it with the anode to see whether there is enough O_2 to oxidize the alloy. Besides that, the reaction between the alloy and the electrolyte and H_2O also need to be taken consideration.

Reply: We appreciate the reviewer for the constructive suggestion. Following this suggestion, we have measured the concentration of O_2 in the O_2-present $ZnSO_4$

electrolyte (~16.59 mg L^{-1}) and calculated the amount of O_2 to be ~8.295×10^{-6} g, or ~2.59×10^{-7} mol, in the sealed cell with electrolyte of 0.5 mL. It means that there is enough O_2 to oxidize the surface atoms of the $Zn_{88}Al_{12}$ alloy with the dimension of 0.5 cm × 0.5 cm × 40 μm, wherein the number of surface atoms is estimated to be ~1.36×10^{-9} mol. In view that the standard equilibrium potential of Al^{3+}/Al (–1.66 V versus SHE) is much lower than that of Zn^{2+}/Zn (–0.76 V versus SHE), the Al surface atoms on the Al lamellas prefer to firstly react with O_2 to form Al_2O_3 during the Zn stripping via the reaction $Zn \rightarrow Zn^{2+} + 2e^-$. Meanwhile, some Zn^{2+} ions take part in the irreversible reaction, $Zn^{2+} + OH^- \rightarrow Zn(OH)_2 + 2e^- \rightarrow ZnO + H_2O$, to form ZnO. Therefore, there may take place reactions: $Zn_{88}Al_{12} + O_2 + H_2O \rightarrow Al_2O_3 + Zn(OH)_2 + Zn^{2+} + e^-$ and $Zn(OH)_2 + 2e^- \rightarrow ZnO + H_2O$.

(4) The author claimed the high coulombic efficiency of the alloy anode; however, no data was demonstrated for the columbic efficiency of the anode.

Reply: We thank the reviewer for the suggestion. The coulombic efficiency of the eutectic $Zn_{88}Al_{12}$ alloy anode has been shown in supplementary **Figure 12**. It demonstrates that the Zn stripping/plating on the eutectic $Zn_{88}Al_{12}$ alloy is highly reversible, with the CE approaching 100 %, during the cycling test for more than 100 cycles.

(5) As for the full cell performance, Figure 5b is a little confusing. How did the author get the specific capacity versus the Scan rate?

Reply: We thank the reviewer for the comment. The specific capacity shown in Figure 5b is calculated according to the CV curves at various scan rates (supplementary **Figure 17a,b**).

(6) Generally, when the loading of the cathode is only 1.0 mg/cm2, which is much less the anode, the electrochemical performance usually relies on the cathode part but anode part. As in many previous reports, Zn foil can also support a good electrochemical performance. So, in this paper, the author should give more strong and supportive data to demonstrate the superiority of the alloy anode, for example, limiting the anode/cathode mass ratio.

Reply: We appreciate the reviewer for the constructive suggestion, following which we have adjusted the anode-to-cathode mass ratio from 40:1 to 3:1. Based on the overall mass of anode and cathode, we have calculated the overall energy densities. The detailed results are shown in supplementary **Figure 19**. The overall energy density of the full $Zn_{88}Al_{12}/K_xMnO_2$ battery can reach ~142 Wh kg^{-1} by lowering the anode-to-cathode mass ratio to 3:1.

Reviewers' comments:

Reviewer #1 (Remarks to the Author):

I am ok with the revised manuscript. The authors addressed the technical issues that I raised quite seriuosly. They provided new data and detailed discussions.

Reviewer #2 (Remarks to the Author):

The authors have tried to address my comment, but there is an important contradiction that needs further explanation. The author claimed that Zn88Al12 alloy electrode exhibit superior oxidation-resistance capability relative to the monometallic Zn electrode, which is from the EIS result. However, from the mechanism illustration in the maintext and the answers to the comment 3 of reviewer 2 and to the comment 7 of reviewer 1, the author believe another metal with lower standard equilibrium potential (Al3+/Al,1.66 V versus SHE) than that Zn2+/Zn (−0.76 V versus SHE) is helpful to form the alloy. The author also claim that the Al surface atoms on the Al lamellas prefer to firstly react with O2 to form Al2O3. Then the question is why the alloy has better oxidation resistance.

Response To Reviewers′ Comments

Reviewer #1 (Remarks to the Author):

I am ok with the revised manuscript. The authors addressed the technical issues that I raised quite seriously. They provided new data and detailed discussions.

Reply: We appreciate the reviewer for his/her positive comments and recommendation for publication in *Nature Communications*.

Reviewer #2 (Remarks to the Author):

The authors have tried to address my comment, but there is an important contradiction that needs further explanation.

Reply: We appreciate the reviewer for his/her positive comment. We also thank the reviewer for the suggestion, following which we have revised the manuscript. The detailed correction is listed below.

(1) The author claimed that $Zn_{88}Al_{12}$ alloy electrode exhibit superior oxidation resistance capability relative to the monometallic Zn electrode, which is from the EIS result. However, from the mechanism illustration in the maintext and the answers to the comment 3 of reviewer 2 and to the comment 7 of reviewer 1, the author believe another metal with lower standard equilibrium potential (Al^{3+}/Al, 1.66 V versus SHE) than that Zn^{2+}/Zn (−0.76 V versus SHE) is helpful to form the alloy. The author also claim that the Al surface atoms on the Al lamellas prefer to firstly react with O_2 to form Al_2O_3. Then the question is why the alloy has better oxidation resistance.

Reply: We appreciate the reviewer for the comment. According to the observations (supplementary **Figure 6**) and the electrochemical measurements (Figure 3 and supplementary **Figure 7**), the eutectic $Zn_{88}Al_{12}$ alloy electrodes indeed exhibit superior oxidation-resistance capability compared with monometallic Zn. Despite the fact that the standard equilibrium potential of Al^{3+}/Al (–1.66 V versus SHE) is lower than that of Zn^{2+}/Zn (–0.76 V versus SHE), these experimental results imply the important role of the Al lamellas in improving the oxidation resistance of $Zn_{88}Al_{12}$ alloy. This is because there forms stable and passive Al_2O_3 surface layer to protect against the further oxidation. The correction have been made in the maintext.

REVIEWERS' COMMENTS:

Reviewer #2 (Remarks to the Author):

I am ok with the revised manuscript.

Response To Reviewer's Comments

Reviewer #2 (Remarks to the Author):

I am ok with the revised manuscript.

Reply: We appreciate the reviewer for his/her positive comments and recommendation for publication in *Nature Communications*.
